# Dynamic CT but Not Optimized Multiphase CT Angiography Accurately Identifies CT Perfusion Target Mismatch Ischemic Stroke Patients

**DOI:** 10.3389/fneur.2019.01130

**Published:** 2019-10-23

**Authors:** Huiqiao Tian, Chushuang Chen, Carlos Garcia-Esperon, Mark W. Parsons, Longting Lin, Christopher R. Levi, Andrew Bivard

**Affiliations:** ^1^Department of Neurology, John Hunter Hospital, University of Newcastle, Newcastle, NSW, Australia; ^2^Department of Neurology, The Royal Melbourne Hospital, University of Melbourne, Melbourne, VIC, Australia

**Keywords:** acute ischemic stroke, CT perfusion, dynamic CTA, multiphase CTA, collaterals

## Abstract

Imaging protocols for acute ischemic stroke varies significantly from center to center leading to challenges in research translation. We aimed to assess the inter-rater reliability of collateral grading systems derived from dynamic computed tomography angiography (CTA) and an optimized multiphase CTA and, to analyze the association of the two CTA modalities with CT perfusion (CTP) compartments by comparing the accuracy of dynamic CTA (dCTA) and optimized multiphase CTA (omCTA) in identifying CT perfusion (CTP) target mismatch patients. Acute ischemic stroke patients with a proximal large vessel occlusion who underwent whole brain CTP were included in the study. Collateral status were assessed using ASPECTS collaterals (Alberta Stroke Program Early CT Score on Collaterals) and ASITN/SIR collateral system (the American Society of Interventional and Therapeutic Neuroradiology/Society of Interventional Radiology) on dCTA and omCTA. Eighty-one patients were assessed, with a median ischemic core volume of 29 mL. The collateral assessment with ASPECTS collaterals using dCTA have a similar inter-rater agreement (K-alpha: 0.71) compared to omCTA (K-alpha: 0.69). However, the agreement between dCTA and CTP in classifying patients with target mismatch was higher compared to omCTA (Kappa, dCTA: 0.81; omCTA: 0.64). We found dCTA was more accurate than omCTA in identifying target mismatch patients with proximal large vessel occlusion.

## Introduction

Collateral circulation supplies blood to the ischemic brain when antegrade blood flow is impaired. There is a strong relationship between collateral circulation and the volume of ischemic core, with good collaterals being associated with a smaller ischemic core (1–3) and also potentially slowing infarct growth (4). This has led some to propose that direct imaging of the core and penumbra with perfusion CT (CTP) can be replaced by collateral assessment on computed tomography angiography (CTA). Multiphase CTA (mCTA) with three imaging acquisitions at different time points during contrast injection offers a limited time-resolved assessment of collateral circulation (5). Dynamic CTA (dCTA) derived from CTP source images contains more time points than mCTA (usually >15). This allows complete tracking of the transit of contrast bolus and may remove one of the major problems with mCTA relating to inability to precisely time the three only acquisition time-points in conjunction with the contrast concentration vs. time curve. Collateral assessment methods such as Alberta Stroke Program Early CT Score (ASPECTS) collateral scores on multiphase CTA (6) or the American Society of Interventional and Therapeutic Neuroradiology/Society of Interventional Radiology (ASITN/SIR) collateral grading system (7) on digital subtraction angiography are well-validated, with both methods showing good correlations with baseline ischemic core volume in a previous study (8). However, these assessments are purely qualitative and suffer from inter-rater variability, whereas measurements with CTP are quantitative and can be fully automated. This is particularly important since the recent trials (9–11) which extended the treatment time windows all selected patients based on CTP imaging, and translation of these results may be limited as not all sites have access to the costly automated imaging analysis technology and rely on other means of patient assessments. Furthermore, quantitative (volumetric) measurements of the ischemic core and penumbra derived from CTP assist in treatment decision-making by identifying patients who are likely to benefit from effective reperfusion (12). The ultimate goal of this study is to investigate the association between collateral assessments on CTAs and CTP compartments, and if dCTA or mCTA can efficiently identify CTP target mismatch patients. The aims of the present study were 2-fold:

1. To test the interrater reliability of ASPECTS and ASITN/SIR collateral systems using dCTA and mCTA in patients with complete proximal large vessel occlusions.
2. To assess the association of collaterals graded on dCTA and mCTA with CTP tissue compartments by examining the concordance of dCTA and CTP at identifying target mismatch patients.

We hypothesize that if mCTA had at least equivalent inter-rater reliability to dCTA at collateral grading and similar concordance to CTP for target mismatch patient identification then one might make a stronger case for replacing CTP with a limited mCTA.

## Methods

### Patients

Patients presenting to the John Hunter Hospital (Newcastle, Australia) between May 2010 and August 2017 with the clinical and radiological diagnosis of an anterior circulation ischemic stroke were retrospectively analyzed. Patients routinely underwent pre-treatment whole brain multimodal CT imaging with non-contrast CT and CTP. At admission patients were assessed using the National Institutes of Health Stroke Scale (NIHSS), while the modified Rankin Scale (mRS) was measured at 90 days post stroke. Patients were treated with intravenous thrombolysis or underwent thrombectomy according to local guidelines and the clinical judgment of the treating physician. For this study, patients were included who had a proximal large vessel occlusion, including internal carotid artery (ICA) terminus T or L type, tandem occlusions, and M1 segment of the middle cerebral artery (MCA, pre-bifurcation segment). The study was approved by the Hunter New England Health District ethics committees.

### Imaging

#### Acute Multimodal CT Protocol

Patients were scanned using Toshiba Aquilion 320-slice CT scanner (Toshiba Medical Systems; Tokyo, Japan). The image acquisition commenced 7 s after the initiation of 40 mL contrast injection at 6 ml/s (Ultravist 370 Bayer HealthcCare; Berlin, Germany). Imaging was acquired at 19-time points over 60 s. The image acquisition consisted of three phases: the first phase was a one frame baseline image (80 kV, 310 mA); the second phase started at 11 s and acquired 13-time points (80 kV, 150/300 mA) with 2 s interval; the third phase started at 40 s and acquired 5-time points (80 kV, 150 mA) with 5 s interval. One gantry rotation time was 0.75 s, and it resulted in 320 axial slices with 0.5 mm thickness. FOV was 220 × 220 mm, and matrix was 512 × 512.

#### Imaging Post-processing and Analysis

CTP images were post-processed using MIStar (Apollo Medical Imaging Technology, Melbourne, Australia). The software automatically selects arterial input function (AIF) from an anterior cerebral artery or middle cerebral artery from the unaffected hemisphere, and venous outflow function from superior sagittal sinus. The AIF was automatically deconvolved using singular value decomposition with delay and dispersion correction (13), and perfusion maps were generated. On CTP maps, the perfusion lesion was defined by delay time >3 s, and the ischemic core was defined by relative cerebral blood flow <30% within the perfusion lesion (14). The penumbral tissue volume was defined as perfusion lesion minus ischemic core. The mismatch was defined as the ratio of perfusion lesion and ischemic core. The target mismatch was defined as ischemic core volume <70 mL, penumbral >15 mL, mismatch ratio >1.8 (15). The ischemic core ratio was defined by the ratio of baseline ischemic core and the baseline perfusion lesion on CTP.

**Figure 1:**
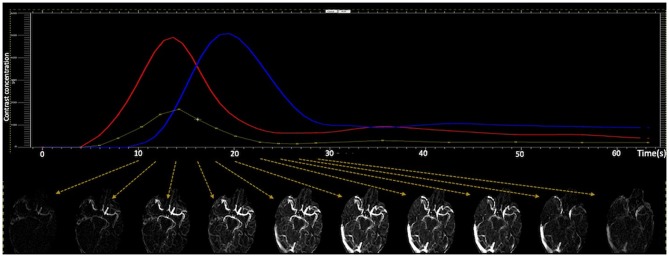
Generating dynamic CTA images. Ten continues CTA images were manually selected from CTP source images, which covers early artery phase, peak arterial phase, late arterial/peak venous phase, and late venous/washout phase.

CTA images were reconstructed from motion corrected CTP source images in axial planes at 24 mm thick-slab maximum intensity projections and 4 mm intervals for each time points on MIStar. For dCTA images, 10 continuous scanning time points were manually selected covering early artery phase, peak arterial phase, late arterial/peak venous phase, and late venous/washout phase (Figure 1). Temporally, the dCTA image for one patient consisted of 10-time points, and spatially, one time point consisted of six 24 mm thick-slab MIP images. Thus, 60 (10 × 6) images constituted one anonymized dynamic CTA image for collateral assessment.

Three-phase CTA (also known as “multiphase CTA”) is not used at our center, therefore, we manually selected three targeted imaging time points from CTP based on arterial and venous contrast vs. time concentration curves (peak arterial phase, peak venous phase and a delayed phase timepoint) (16). Peak arterial phase (the first phase) was selected at the imaging time point with maximum enhancement on the arterial input function curve; peak venous phase (the second phase) was selected at the time point with maximum enhancement on the venous outflow (VOF) curve, which was an 8 ± 2 s delay from the peak arterial phase; and the delayed phase (the third phase) was 8 ± 2 s delay from the peak venous phase. These three-phase CTA we termed “optimized multiphase CTA” (omCTA), because the imaging time points were directly selected from the peak arterial and venous phase, whereas the time interval between the three phases in conventional multiphase CTA is set at 8 s and may not necessarily reflect peak arterial and venous contrast (rather, the phases are set a certain time after contrast injection) (6). The final omCTA images for one patient consist of three phases in temporal, and six 24 mm thick-slab MIP images for each phase. Thus, 18 (3 × 6) images constituted one anonymized omCTA image.

#### Collateral Assessments

Collateral assessments on dCTA and omCTA were performed by four raters: a senior stroke imaging neurologist (C.L., with more than 20 years of experience), a stroke imaging neurologist (C.G-E., with more than 5 years of experience) and two stroke imaging researchers (C.C. and H.T. with more than 5 and 3 years of experience, respectively). Raters were blinded to patient's clinical information and each other's findings. Collateral status was evaluated with ASPECTS collaterals (6) and ASITN/SIR (7), both reported high correlation with early infarct core (8). Both collateral systems ranged from no visible collateral vessel (Grade 0) through lower grades for few collateral vessel with delay to rapid and normal collateral flow (Grade 5 at ASPECTS collaterals and grade 4 at ASITN/SIR, Supplementary Tables 1, 2).

A training document (entitled as “suggested standards for collateral assessment on dCTA and omCTA,” Supplementary Figure 1) was created with an absolute agreement on collateral scores of the raters under the supervision of C.L. The document contained representative angiographic images for each score of the two scoring systems and imaging modalities. The four raters carefully reviewed the training document for a better visualized learning, and then performed collateral assessments. Any disagreement was resolved by consensus meeting, and the final collateral scores of the 81 patients on each grading system and imaging modality were recorded as gold standards.

To investigate if the training document improves the inter-rater agreement on collateral assessment, two raters (C.G-E. and C.C.) were asked to assess the collaterals based on the methods described in Supplementary Tables 1, 2 prior to the release of training document. Then, the trainees were asked for a second round of collateral assessments for the same patient cohort after training. The two collateral assessments separated in time by 6 weeks, and the results used for an analysis on training effects.

### Statistical Analysis

Statistical analysis was performed using STATA (v13.0; StataCorp LP, College Station, TX). Patient demographic and clinical characteristics were summarized, and CTP tissue compartments were examined against final ASPECTS collateral score assessed on dCTA. Descriptive results were presented as mean ± standard deviation (SD) or median with 1st and 3rd quantiles (Q1-Q3). (1) To assess the inter-rater reliability of the four raters after training, we used weighted Krippendorff's alpha; (2) To assess the possible learning effects, we calculated the weighted Krippendorff's alpha of the collateral scores rated by the trainees and the scores defined as gold standards, before and after training; (3) To assess the relationships between collateral scores and CTP tissue compartments (perfusion lesion, baseline ischemic core, and core ratio), we used Spearman's rho; (4) To examine the ASPECTS collateral score cutoff point for classifying patients with target mismatch on CTP (15), we performed a receiver operating characteristic (ROC) analysis, and used DeLong method to compare the area under the ROC; (5) We examined the intermodality agreement of dCTA and CTP, and the agreement of omCTA and CTP in classifying patient with target mismatch using Cohen's kappa. Target mismatch was a binary variable (yes/no); therefore, the six-point ASPECTS collateral scale were dichotomized into good and poor collaterals based on the cutoff point carried out from (4).

To interpret inter-rater and inter-modality reliability results, we adopted the Landis and Koch approach. Krippendorff's-alpha ≥ 0.8: almost perfect agreement; 0.61–0.80: substantial agreement, 0.41–0.60: moderate agreement; 0.21–0.40: fair agreement; and ≤0.2 slight agreement. The statistical significance level was set at *P* < 0.05.

## Results

During the study period, 346 patients presenting to the hospital who had an anterior circulation stroke were assessed both clinically and radiologically. Of the 346 patients, 134 patients were excluded because they had an incomplete follow-up clinical data, 68 because of an incomplete follow-up MRI, and 18 because of imaging artifacts. Of the remaining 126 patients, 26 had distal MCA vessel occlusions, and 19 partial or no vessel occlusions. Therefore, 81 patients with complete occlusion in the ICA or the M1-MCA were the final patient cohort. Patients included in this study had a moderate to severe strokes, with the median NIHSS score 18 (Q1-Q3 14–20) and the median ischemic core volume of 29 mL (Q1-Q3 10–77, Table 1).

**Table 1:** Patient clinical and imaging characteristics grouped by treatment received.

	**All patients**	**Endovascular therapy**	**r-tPA treated**
Number of patients, *n* (%)	81 (100)	18 (22)	47 (58)
Mean age, y(SD)	72 (13)	67 (14)	72 (14)
Median Time to scan, min (Q1–Q3)	116 (91–140)	91 (66–120)	120 (92–140)
Median baseline NIHSS (Q1-Q3)	18 (14–20)	18 (14–19)	18 (14–21)
Median baseline perfusion lesion, mL (Q1–Q3)	138 (104–201)	135 (107–168)	141 (107–209)
Median baseline ischemic core, mL (Q1–Q3)	29 (10–77)	28 (15–54)	29 (8–46)

### Inter-rater Reliability

**Figure 2:**
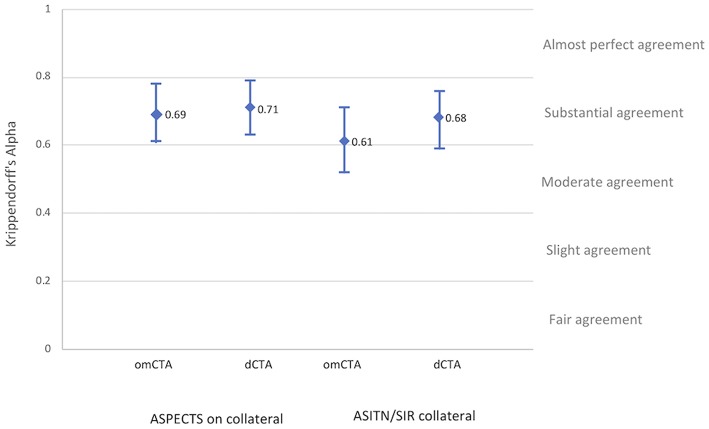
Inter-rater reliability. Inter-rater reliability of the four raters after training for each angiographic imaging modality and collateral grading systems.

Collateral assessments of the four raters (C.L., C.G-E., C.C. and H.T.) on both dCTA and omCTA reached substantial agreement. Among the two collateral scoring systems on the two CTA modalities, the ASITN/SIR system on omCTA had the lowest inter-rater reliability (K-alpha 0.61), and ASPECTS collateral scoring system on dCTA had the highest reliability (K-alpha 0.71, Figure 2).

### Training Effects

Before training, the raters (C.G-E. and C.C.) had a low agreement with the gold standard on both dCTA (K-alpha: 0.59 for ASPECTS collaterals and 0.50 for ASITN/SIR), and on omCTA (K-alpha: 0.54 for ASPECTS collateral and 0.46 for ASITN/SIR). After training, the raters achieved a substantial agreement in collateral assessments with the gold standard on dCTA (K-alpha: 0.73 for ASPECTS collateral and 0.66 for ASITN/SIR), however the overall agreement was lower on omCTA (K-alpha: 0.60 with ASPECTS collateral and 0.63 with ASITN/SIR, Supplementary Figure 2) compared to dCTA.

### Association of omCTA vs. dCTA With CTP Tissue Compartments

ASPECTS collateral score had a stronger association with ischemic core on dCTA (Spearman's ρ = −0.79, *P* < 0.001, Table 2, Supplementary Figure 3) compared to omCTA (Spearman's ρ = −0.71, *P* < 0.001). Furthermore, there was a negative correlation between ischemic core ratio and collateral status (Spearman's ρ raged from −0.61 to −0.71, all *P* < 0.001, Table 2).

**Table 2:** The correlation of collateral scores with CTP compartments.

	**Perfusion lesion (DT > 3 s)**	**Ischemic core volume**	**Core-ratio**
**ASPECTS collateral**			
dCTA	−0.71	−0.79	−0.71
omCTA	−0.68	−0.71	−0.61
**ASITN/SIR**			
dCTA	−0.69	−0.76	−0.67
omCTA	−0.65	−0.72	−0.65

*P-values of all Spearman's ρ < 0.001*.

### Inter-modality Agreement

**Figure 3:**
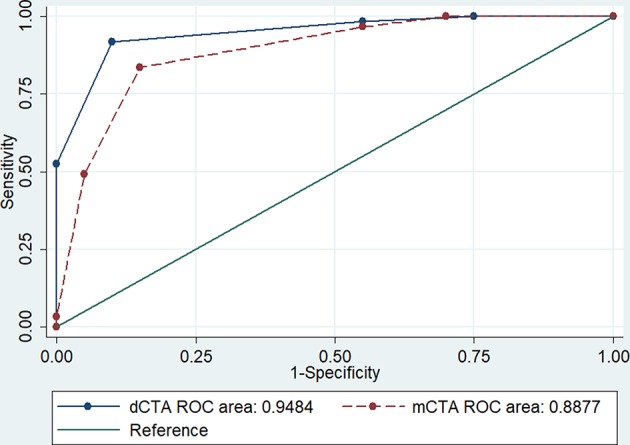
The receiver operating characteristic analysis. The ASPECTS collateral scale classifies well patients with target mismatch on both dCTA and omCTA. The cutoff point with best prediction is score 3, with 0.92 sensitivity and 0.95 for specificity with dCTA; and 0.84 sensitivity and 0.89 for specificity with omCTA. AUC, area under the curve.

A large proportion of patients (96%) who had an ASPECTS collateral score of 3 were also classified as having target mismatch on CTP. Comparatively, patients with an ASPECTS collateral score of 2 had larger median baseline ischemic core (score 3: 29 mL, Q1-Q3 21–42; score 2: 84 mL, Q1-Q3 48–103, *p* < 0.001) and smaller ischemic core ratio (score 3: 0.22 ± 0.11; score 2: 0.41 ± 0.21, *p* = 0.002, Table 3). The ROC analysis identified score 3 was the cutoff point of ASPECTS collaterals with the best prediction of target mismatch (area under the curve, dCTA: 0.95; omCTA: 0.89, *p* = *0.076*, Figure 3). Therefore, scores 3–5 were considered as “good collaterals,” score 0–2 were “poor collaterals.”

**Table 3:** Patient clinical and imaging characteristics grouped by ASPECTS collateral scores.

	**ASPECTS collateral scores on dCTA**					
	**Score 5**	**Score 4**	**Score 3**	**Score 2**	**Score 1**	**Score 0**
Number of patients, *n* (%)	2 (3)	30 (37)	26 (32)	13 (16)	5 (6)	5 (6)
Median perfusion lesion, mL(Q1–Q3)	32 (19–45)	104 (72–130)	137 (120–168)	189 (169–224)	242 (225–249)	293 (232–411)
Median baseline ischemic core, mL(Q1–Q3)	8 (1–17)	9 (4–21)	29 (21–42)	84 (48–103)	114 (94–170)	206 (151–220)
Mean Baseline ischemic core ratio (SD)	0.20 (0.23)	0.11 (0.10)^*^	0.22 (0.11)	0.41 (0.21)^**^	0.49 (0.13)	0.66 (0.13)
Target mismatch, *n* (%)	2 (100)	30 (100)	25 (96)	4 (31)	1 (20)	0 (0)
Follow-up DWI infarct, mL (Q1–Q3)	10 (2–18)	16 (9–35)	46 (12–89)	97 (22–123)	154 (124–250)	208 (195–256)
Median mRS at 90-day (Q1–Q3)	3 (1–5)	2 (1–4)	2 (1–5)	5 (1–6)	6 (5–6)	6 (6–6)

* The mean ischemic core ratios between score 4 and 3 are different: P = 0.005.

** *The mean ischemic core ratios between score 3 and 2 are different: P = 0.002*.

Dynamic CTA and CTP reached an almost perfect agreement in classifying target mismatch patients (κ = 0.81, 93% agreement, 7 of 81 patients were misclassified). However, the omCTA and CTP only had a moderate agreement (κ = 0.64, 85% agreement, 13 of 81 patients were misclassified).

## Discussion

This study introduced dynamic CTA and optimized multiphase CTA, and dCTA showed improved reliability on the assessment of collaterals compared to omCTA. We found dCTA had high concordance (κ = 0.81) in identifying patients with CTP target mismatch, while the omCTA was moderate (κ = 0.64). Next, inter-rater agreement of collateral assessments in acute stroke patients with a large vessel occlusion on dCTA or omCTA was modest, although the agreement was substantially improved with training on dCTA, and to a lesser degree with omCTA. Lastly, collateral scores assessed on dCTA and omCTA were strongly correlated with the baseline perfusion lesion and ischemic core volume.

Our results are in line with the previous finding that most patients who had a proximal large vessel occlusion with a moderate-to-good collateral circulation on the ASPECTS collateral score are more likely to have a large penumbral volume (17). For example, the ESCAPE trial (Endovascular Treatment for Small Core and Anterior Circulation Proximal Occlusion with Emphasis on Minimizing CT to Recanalization Times) used collateral assessments on mCTA for endovascular thrombectomy patient selection (5), and most patients included in the ESCAPE trial who underwent CTP imaging had a penumbral pattern (17). This is because patient with a good collateral circulation are more likely to have a large penumbra lesion with a corresponding small ischemic core and so were very likely to have target mismatch. Furthermore, a recent study found that collateral score of >3 on mCTA better identified patients who met CTP target mismatch criteria compared to single-phase CTA (18). Similarly, we found collateral score of >3 was the cut-off point of identifying target mismatch patients on both omCTA and dCTA, with dCTA having a higher sensitivity and specificity, and a lower rate of misclassification (7 vs. 15% patients were misclassified on dCTA and omCTA, respectively). Although the area under the curves are not substantially different between the two CTAs for predicting target mismatch patients (area under the curve, dCTA: 0.95; omCTA: 0.89, *p* = 0.076), however, there is a trend that dCTA is more accurate than omCTA. Therefore, the strong correlation of collateral scores assessed on dCTA with the CTP compartments and the improved inter-rater agreement of collateral assessment after training on dCTA compared to omCTA suggest that dCTA might be a more reliable imaging tool for assessing acute ischemic stroke patients when CTP post-processing software is not available.

In the present study, we optimized the conventional mCTA by manually selecting the three scanning time points to avoid missing arterial and venous peaks of the contrast bolus. That is, the omCTA that used in this study cannot be assumed to be equivalent to the conventional mCTA. However, optimizing the scanning time did not appear to improve the inter-rater reliability of omCTA compared to dCTA. Also, dCTA provided a broader variation for better and more concise collateral assessments for readers than omCTA. The differences in training effects between the dCTA and omCTA suggests that the variability of collateral scores is larger when the CTA modality contains low temporal resolution.

The acquisitions of CTP and dCTAs are acquired simultaneously, but the data they display is quite different, with CTP providing summary maps which reduces the need for interpretation. Furthermore, perfusion maps generated by an automated software offer objective volumetric values and avoid the assessment variation of different raters or evaluation environment. Importantly as well, assessing collateral status on CTA modalities usually require an extra 1–2 min compared to reading the automatically generated perfusion maps on CTP. When the collaterals are between moderate and poor, even the experienced raters had to compare the patient CTA imaging with the training material to ensure a reliable result. For this study, the raters were able to complete collateral assessments at a research environment, while in the clinical setting where there is significant time pressure, the automated CTP post-processing has substantial advantages since it removes qualitative assessment.

Some study limitations need to be acknowledged. (1) This is a single center study, and all included patients had a complete anterior proximal large vessel occlusion. Therefore, the relationship between the collateral scores and the CTP tissue compartments was studied specifically to this cohort of patients. That is, the strong relationships between the dCTA and CTP imaging modalities may not exist in patients with distal vessel occlusions or posterior circulation occlusions; (2) digital subtraction angiography and single-phase CTA were not available for every patient and were not included in our study; (3) a high temporal resolution of CTP may improve the simulation of mCTA (19), however it is not possible to achieve in this retrospective analysis; (4) although dCTA accurately identified CTP target mismatch patients, however, assessing collaterals on dCTA may not replace the measurements from CTP because dCTA misclassified 7 out of 81 patients. Also, CTP provides more information than simply the presence of target mismatch or not for an individual patient, such as the extent of salvageable tissue or the degree of hypoperfused tissue, and this should be taken into consideration when interpreting the results; (5) Lastly, it is important to acknowledge that the treatment thresholds for thrombectomy and thrombolysis futility are currently unknown, and even with good or poor collateral circulation some patients may benefit from therapy still and this study is not intended to alter the clinical treatment protocols but inform on the imaging techniques used.

In conclusion, we introduced collateral assessment methods on dCTA and examined it in two aspects. We found dCTA offers a more reliable assessment on collaterals and has a stronger association with CTP tissue compartments than omCTA. Additionally, dCTA is a reader-dependent imaging tool and might require more time for clinician's input compared to CTP, however, our results support that dCTA is a reliable imaging modality for assessing collaterals or identifying CTP target mismatch patients. Therefore, dCTA is encouraged for routine clinical practice when CTP post-processing software is not available.

## Data Availability Statement

Anonymized data used in this study will be shared on request to the corresponding author.

## Ethics Statement

The studies involving human participants were reviewed and approved by Hunter New England Health District ethics committees. The patients/participants provided their written informed consent to participate in this study.

## Author Contributions

HT contributed to the study concept and design, data analysis, and article drafting. CC contributed to the study design, data analysis, and article revising. CG-E contributed to data analysis and article revising. MP and LL contributed to data acquisition and article revising. CL contributed to the study concept and design, data acquisition, data analysis, and article revising. AB contributed to the study concept and design, data acquisition, and article revising.

### Conflict of Interest

The authors declare that the research was conducted in the absence of any commercial or financial relationships that could be construed as a potential conflict of interest.
