# Supplementary material for: Dynamic CT but Not Optimized Multiphase CT Angiography Accurately Identifies CT Perfusion Target Mismatch Ischemic Stroke Patients
Source: Front Neurol. 2019 Oct 23;10:1130. doi: 10.3389/fneur.2019.01130 (PMC6819495; doi:10.3389/fneur.2019.01130)
Supplement: Supplementary file 1 [file Table_1.DOCX]

**Supplementary document**

**Table 1.** Collateral score using dynamic CT angiography

| ASPECTS on collaterals | Collateral – ASITN/SIR |
| --- | --- |
| **Score 0:** When compared with the asymptomatic contralateral hemisphere, there are no vessels visible in any time frame within the ischemic vascular territory | **Grade 0:** no collateral vessels visible |
| **Score 1:** When compared with the asymptomatic contralateral hemisphere, there are just a few vessels visible in any time frame within the occluded vascular territory | **Grade 1:** slow collateral blood flow to the periphery of the ischemic site with persistence of some of the defect |
| **Score 2:** When compared with the asymptomatic contralateral hemisphere, there is a delay of 4 frames (8 seconds) in filling in of peripheral vessels and decreased prominence and extent or a 2-frame delay and some ischemic regions with no vessels | **Grade 2:** rapid collateral blood flow to the periphery of ischemic site with persistence of some of the defect |
| **Score 3:** When compared with the asymptomatic contralateral hemisphere, there is a delay of 4 frames in filling in of peripheral vessels or there is a 2-frame delay and significantly reduced number of vessels in the ischemic territory | **Grade 3:** collateral flow with slow but complete angiographic blood flow of the ischemic bed by the late venous phase |
| **Score 4:** When compared with the asymptomatic contralateral hemisphere, there is a delay of 2-frames in filling in of peripheral vessels, but prominence and extent is the same | **Grade 4:** complete and rapid collateral blood flow to the vascular bed in the entire ischemic territory by retrograde perfusion |
| **Score 5:** When compared with the asymptomatic contralateral hemisphere, there is no delay and normal or increased prominence of pial vessels/normal extent within the ischemic territory in the symptomatic hemisphere |  |

*Rapid collateral blood flow = filling within 2 frames (<4 seconds)

**Table 2.** Collateral score using multiphase CT angiography

| ASPECTS on collaterals | Collateral – ASITN/SIR |
| --- | --- |
| **Score 0:** When compared with the asymptomatic contralateral hemisphere, there are no vessels visible in any phase within the ischemic vascular territory | **Grade 0:** no collateral vessels visible |
| **Score 1:** When compared with the asymptomatic contralateral hemisphere, there are just a few vessels visible in any phase within the occluded vascular territory | **Grade 1:** slow collateral blood flow to the periphery of the ischemic site with persistence of some of the defect |
| **Score 2:** When compared with the asymptomatic contralateral hemisphere, there is a delay of two phases in filling in of peripheral vessels and decreased prominence and extent or a one-phase delay and some ischemic regions with no vessels | **Grade 2:** rapid collateral blood flow to the periphery of ischemic site with persistence of some of the defect |
| **Score 3:** When compared with the asymptomatic contralateral hemisphere, there is a delay of two phases in filling in of peripheral vessels or there is a one-phase delay and significantly reduced number of vessels in the ischemic territory | **Grade 3:** collateral flow with slow but complete angiographic blood flow of the ischemic bed by the late venous phase |
| **Score 4:** When compared with the asymptomatic contralateral hemisphere, there is a delay of two phases in filling in of peripheral vessels, but prominence and extent is the same | **Grade 4:** complete and rapid collateral blood flow to the vascular bed in the entire ischemic territory by retrograde perfusion |
| **Score 5:** When compared with the asymptomatic contralateral hemisphere, there is no delay and normal or increased prominence of pial vessels/normal extent within the ischemic territory in the symptomatic hemisphere |  |

*Rapid collateral flow = flow appearing within the arterial phase
