# Supplementary material for: Dynamic CT but Not Optimized Multiphase CT Angiography Accurately Identifies CT Perfusion Target Mismatch Ischemic Stroke Patients
Source: Front Neurol. 2019 Oct 23;10:1130. doi: 10.3389/fneur.2019.01130 (PMC6819495; doi:10.3389/fneur.2019.01130)
Supplement: Supplementary file 2 [file Presentation_1.PPTX]

## Slide 1
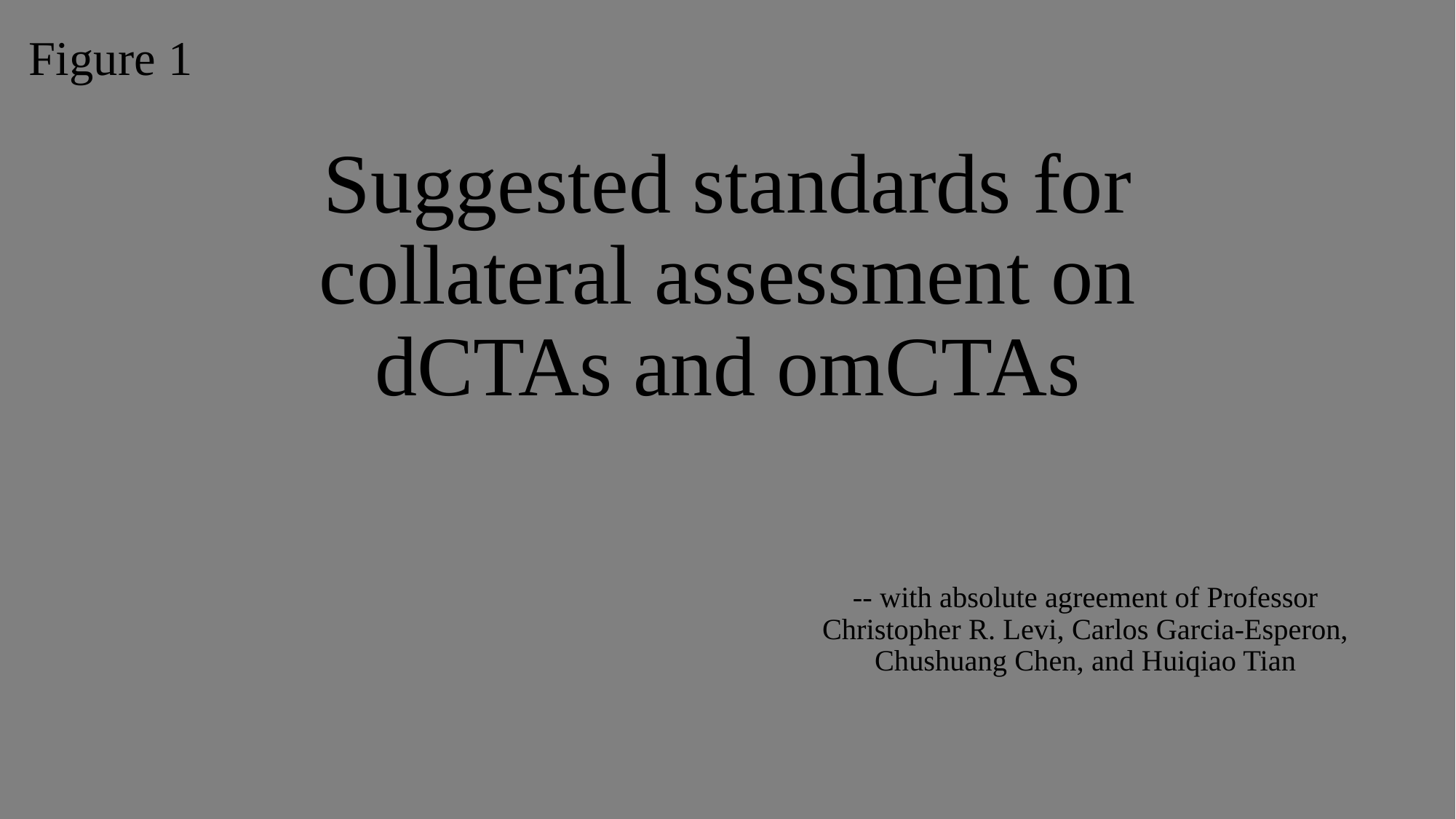

Figure 1
# Suggested standards for collateral assessment on dCTAs and omCTAs
-- with absolute agreement of Professor Christopher R. Levi, Carlos Garcia-Esperon, Chushuang Chen, and Huiqiao Tian

## Slide 2
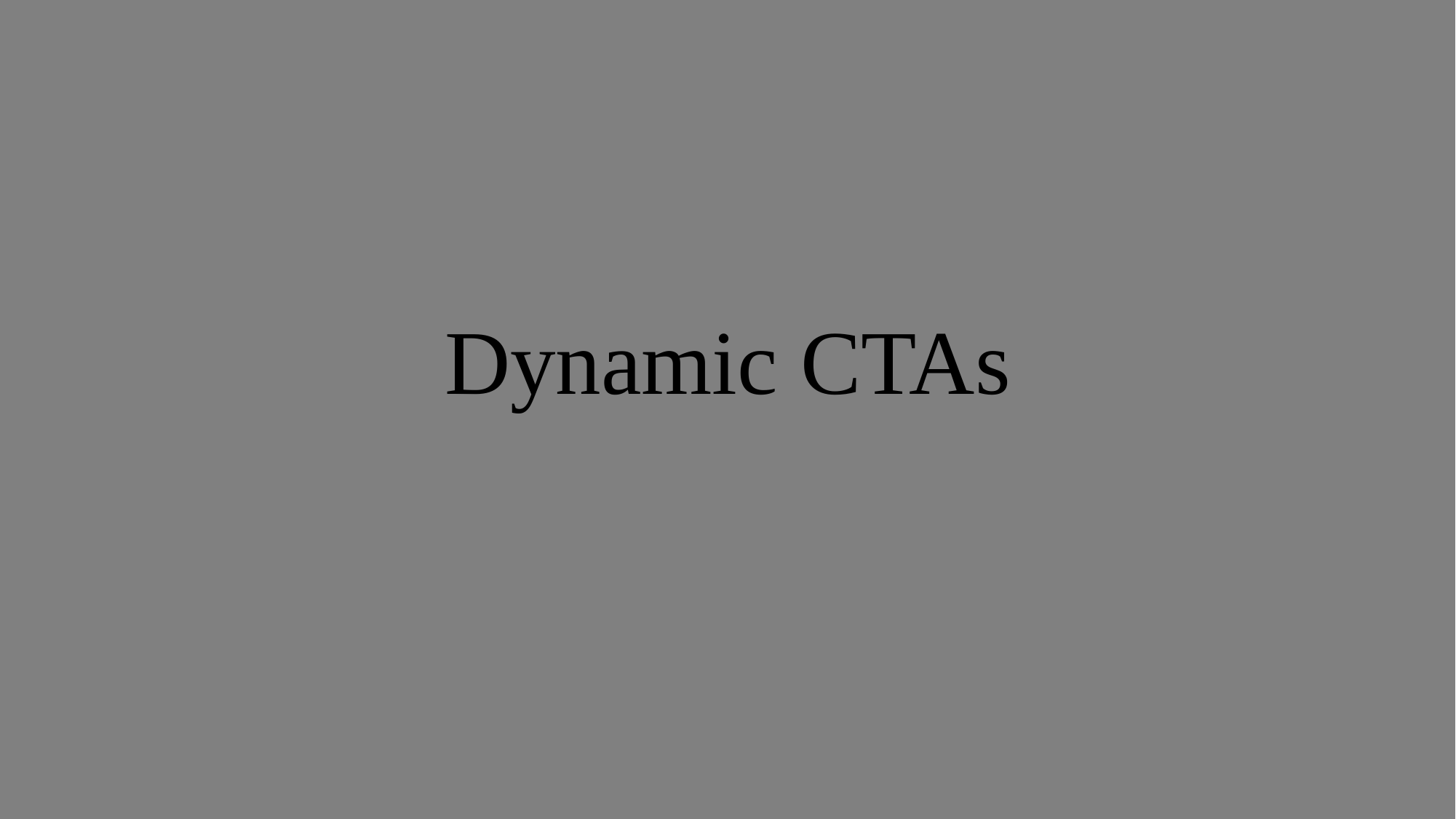

# Dynamic CTAs

## Slide 3
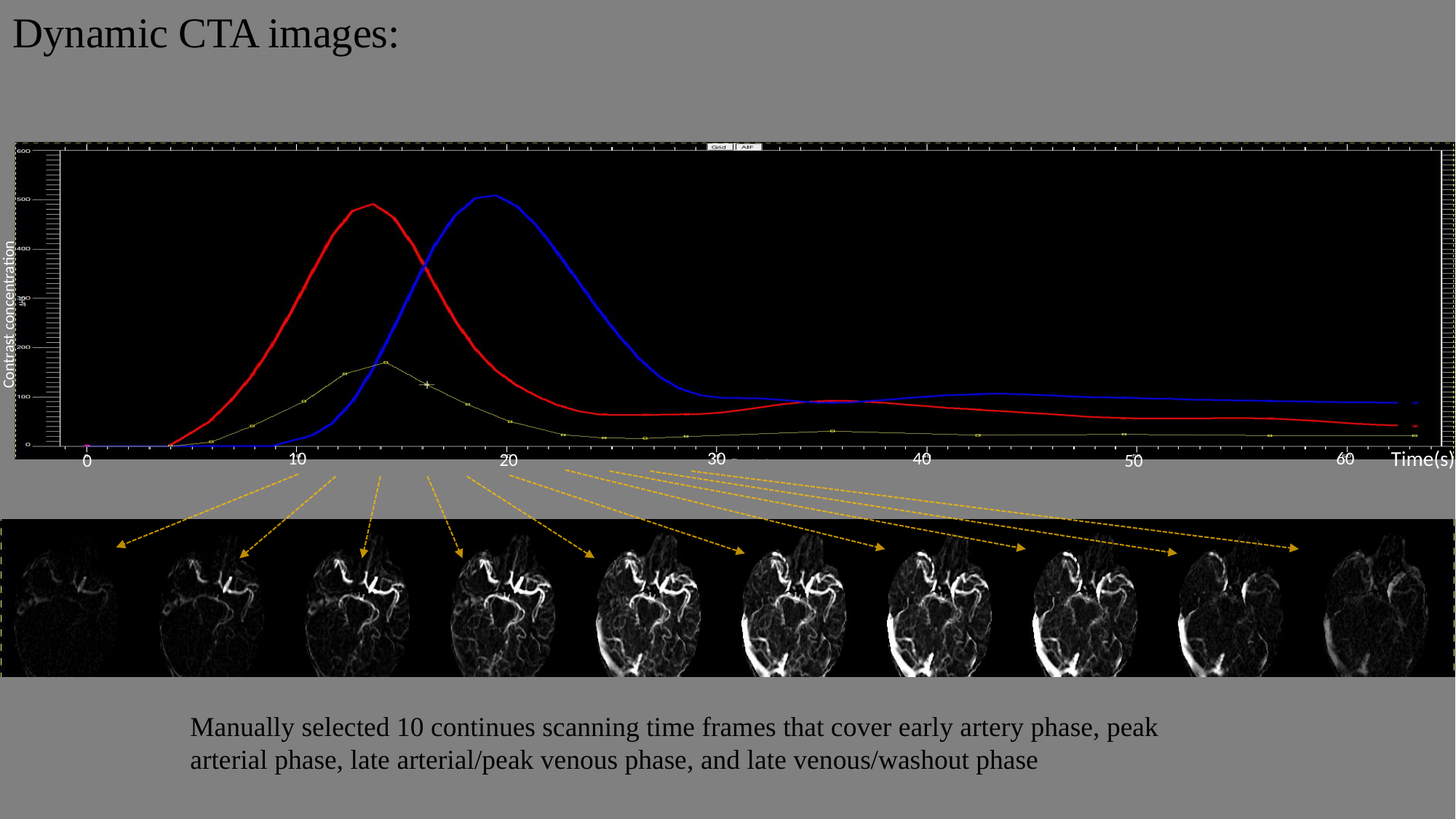

Dynamic CTA images:
Contrast concentration
Time(s)
10
40
60
30
20
0
50
Manually selected 10 continues scanning time frames that cover early artery phase, peak arterial phase, late arterial/peak venous phase, and late venous/washout phase

## Slide 4
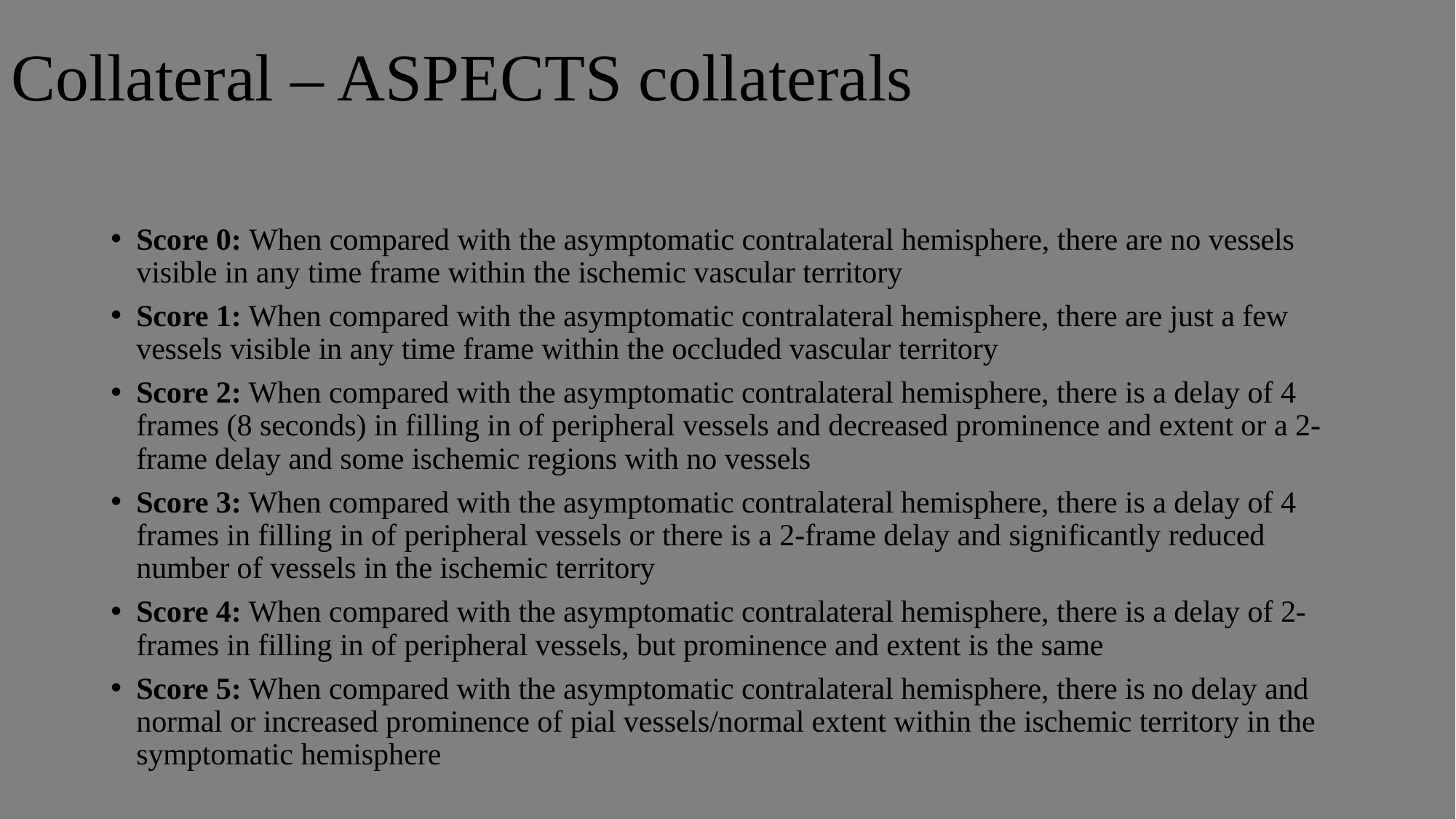

# Collateral – ASPECTS collaterals
Score 0: When compared with the asymptomatic contralateral hemisphere, there are no vessels visible in any time frame within the ischemic vascular territory
Score 1: When compared with the asymptomatic contralateral hemisphere, there are just a few vessels visible in any time frame within the occluded vascular territory
Score 2: When compared with the asymptomatic contralateral hemisphere, there is a delay of 4 frames (8 seconds) in filling in of peripheral vessels and decreased prominence and extent or a 2-frame delay and some ischemic regions with no vessels
Score 3: When compared with the asymptomatic contralateral hemisphere, there is a delay of 4 frames in filling in of peripheral vessels or there is a 2-frame delay and significantly reduced number of vessels in the ischemic territory
Score 4: When compared with the asymptomatic contralateral hemisphere, there is a delay of 2-frames in filling in of peripheral vessels, but prominence and extent is the same
Score 5: When compared with the asymptomatic contralateral hemisphere, there is no delay and normal or increased prominence of pial vessels/normal extent within the ischemic territory in the symptomatic hemisphere

## Slide 5
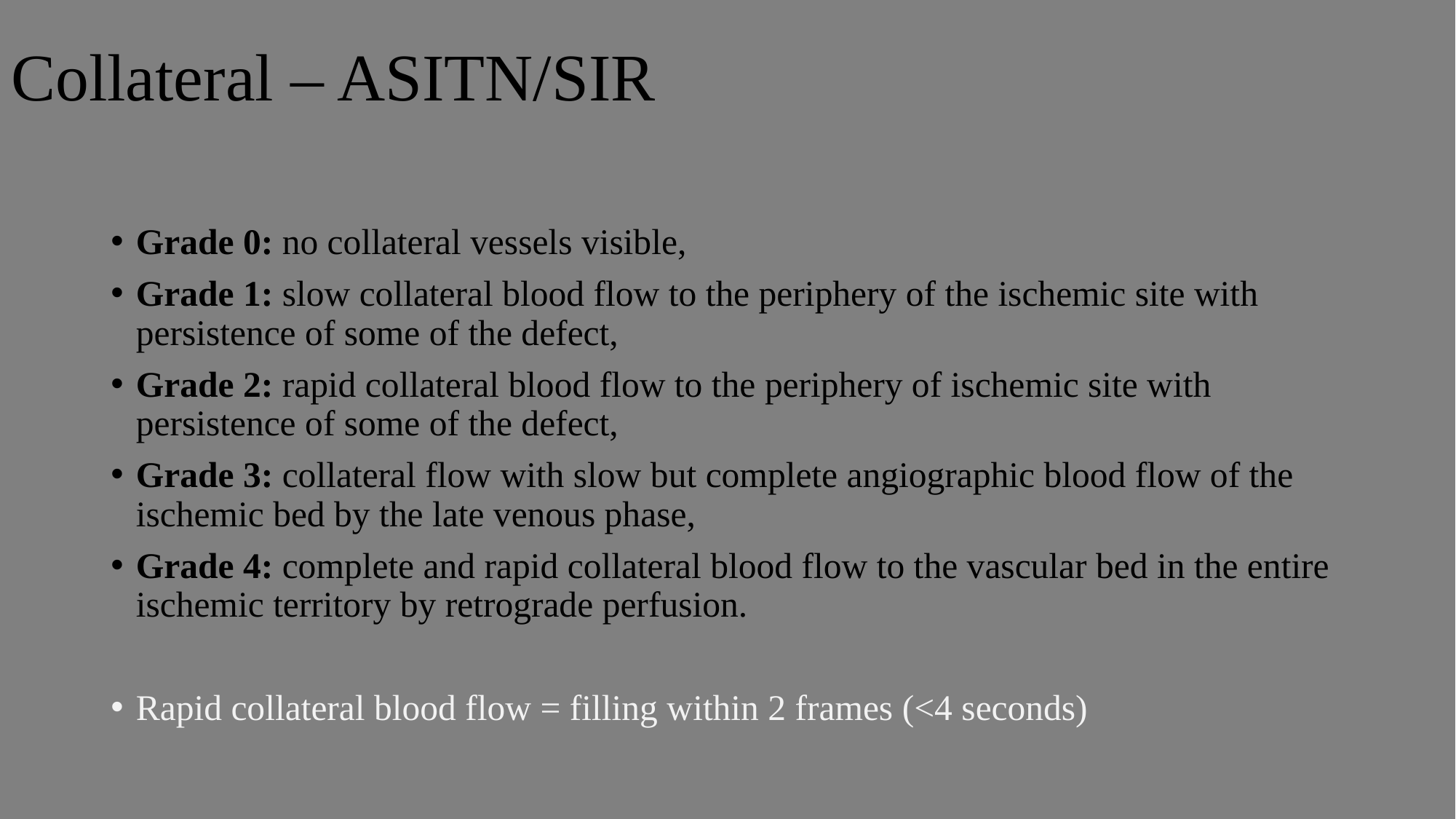

# Collateral – ASITN/SIR
Grade 0: no collateral vessels visible,
Grade 1: slow collateral blood flow to the periphery of the ischemic site with persistence of some of the defect,
Grade 2: rapid collateral blood flow to the periphery of ischemic site with persistence of some of the defect,
Grade 3: collateral flow with slow but complete angiographic blood flow of the ischemic bed by the late venous phase,
Grade 4: complete and rapid collateral blood flow to the vascular bed in the entire ischemic territory by retrograde perfusion.
Rapid collateral blood flow = filling within 2 frames (<4 seconds)

## Slide 6
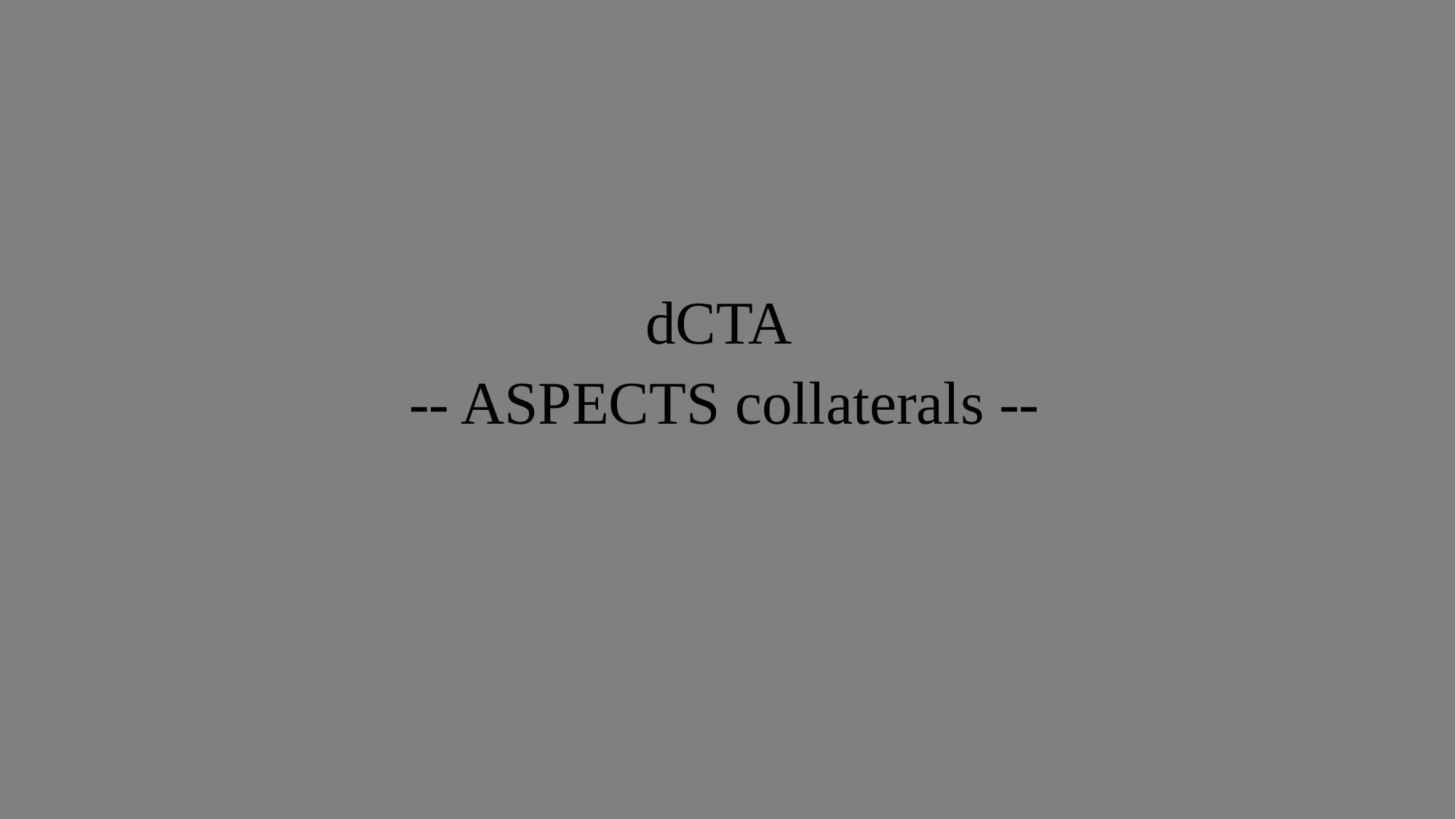

dCTA
-- ASPECTS collaterals --

## Slide 7
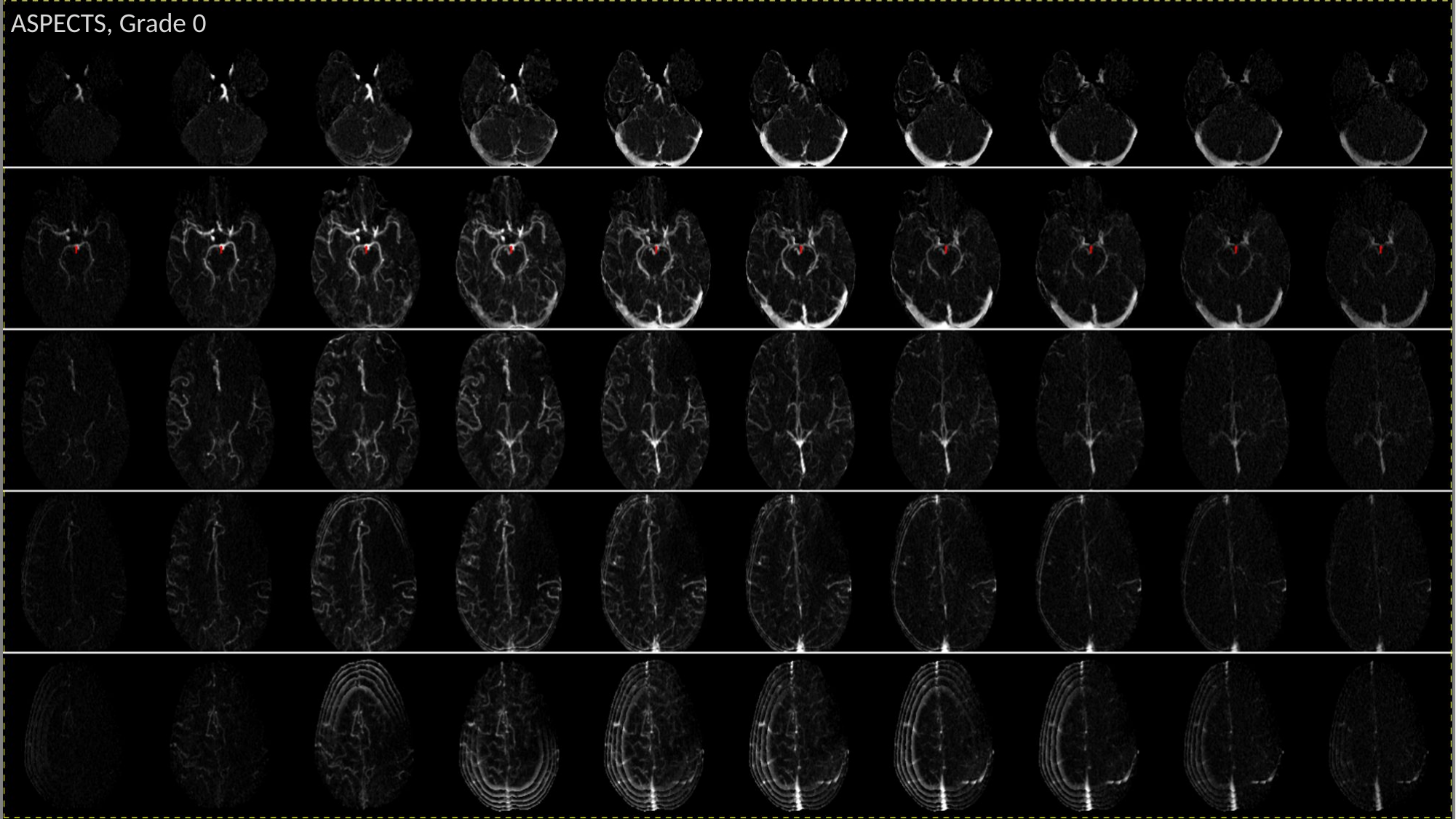

ASPECTS, Grade 0

## Slide 8
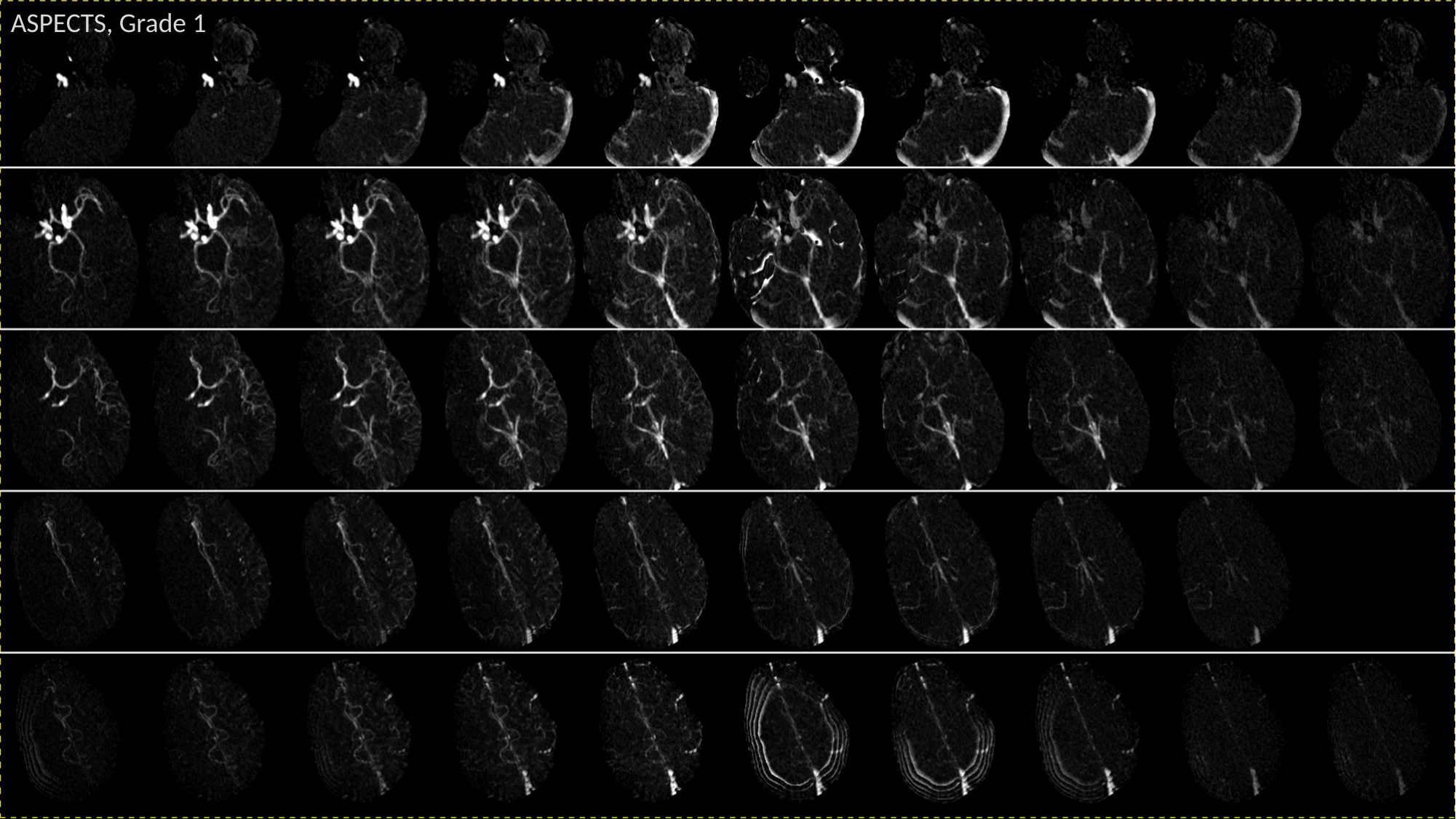

ASPECTS, Grade 1

## Slide 9
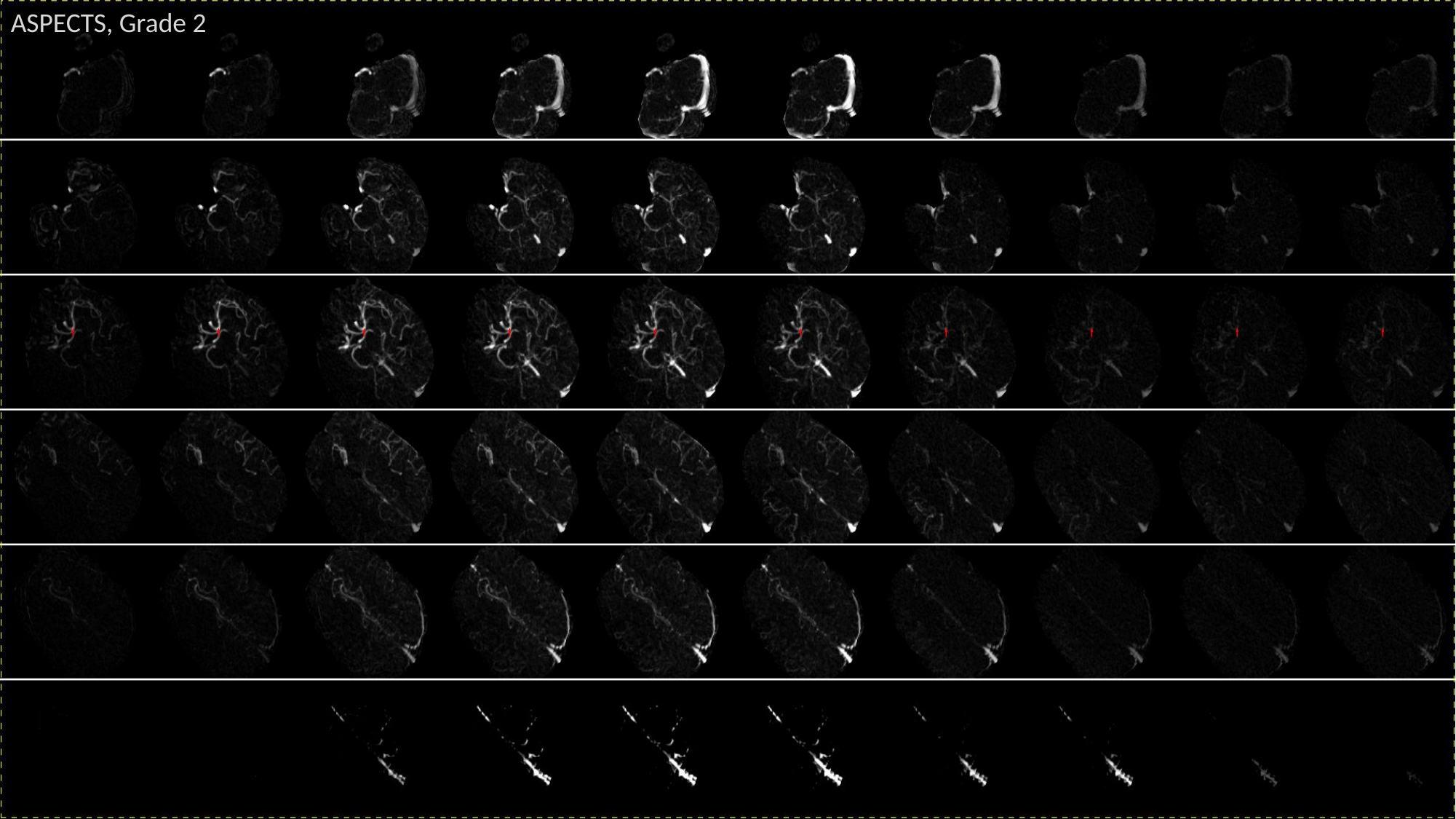

ASPECTS, Grade 2

## Slide 10
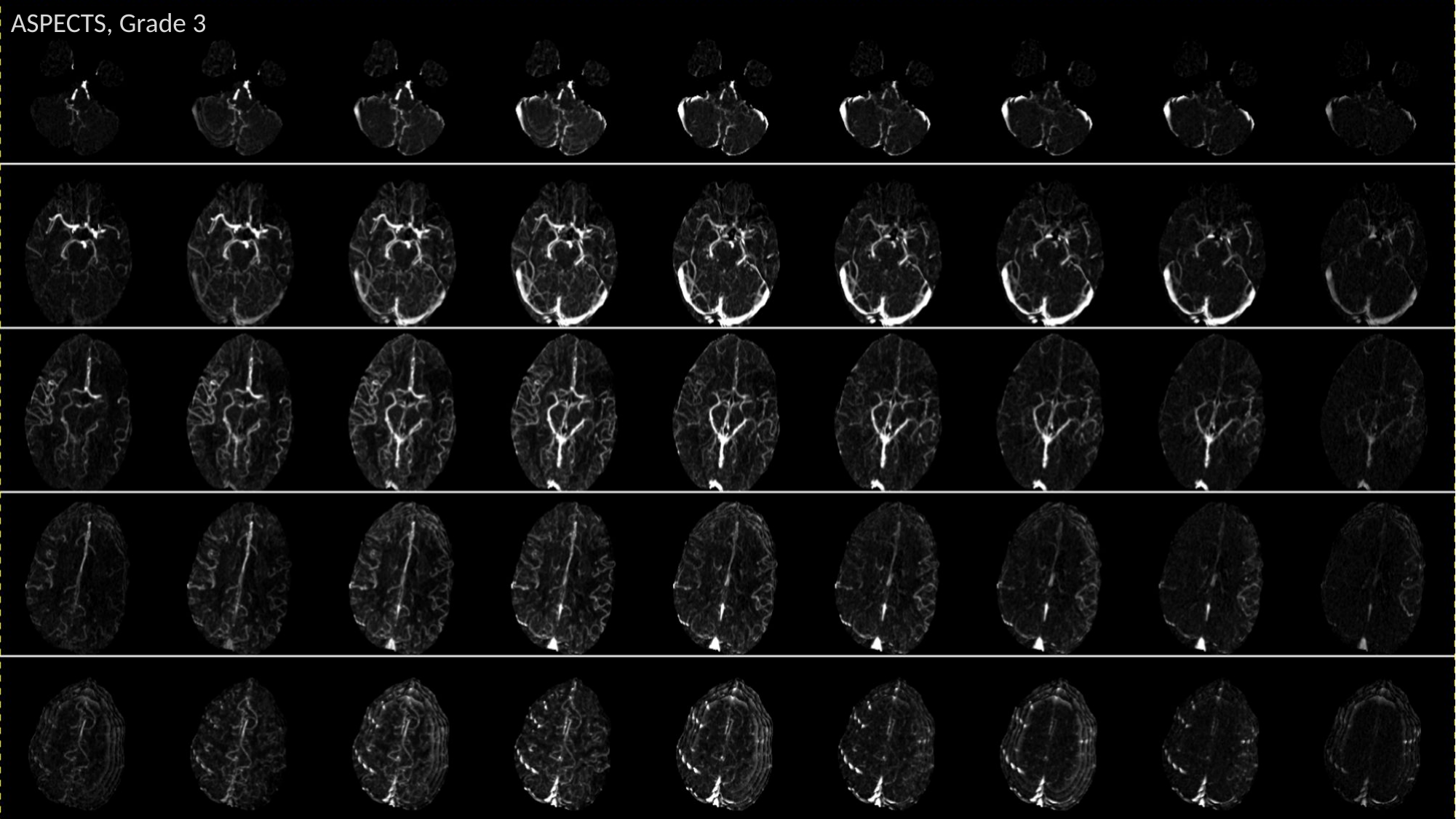

ASPECTS, Grade 3

## Slide 11
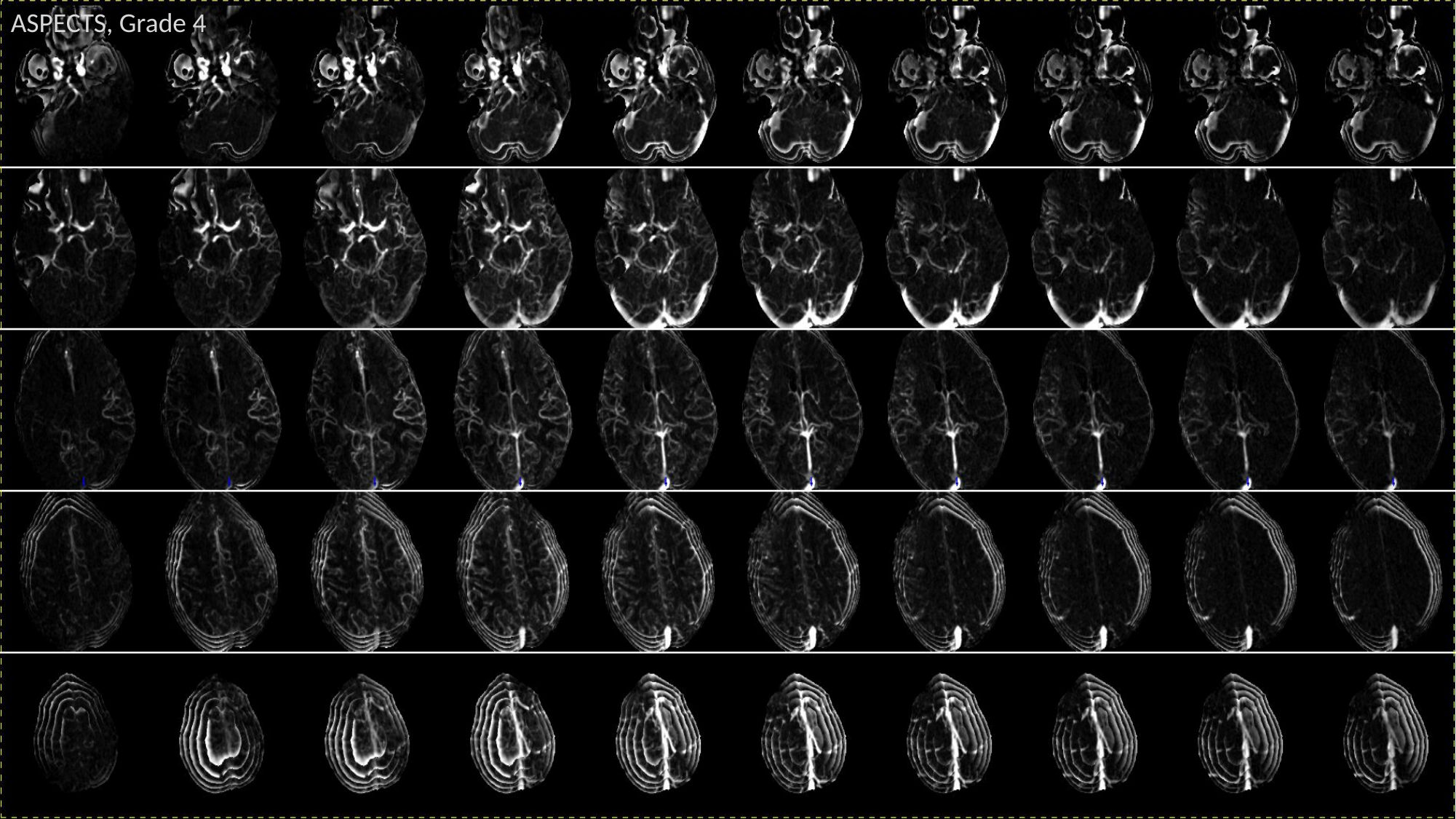

ASPECTS, Grade 4

## Slide 12
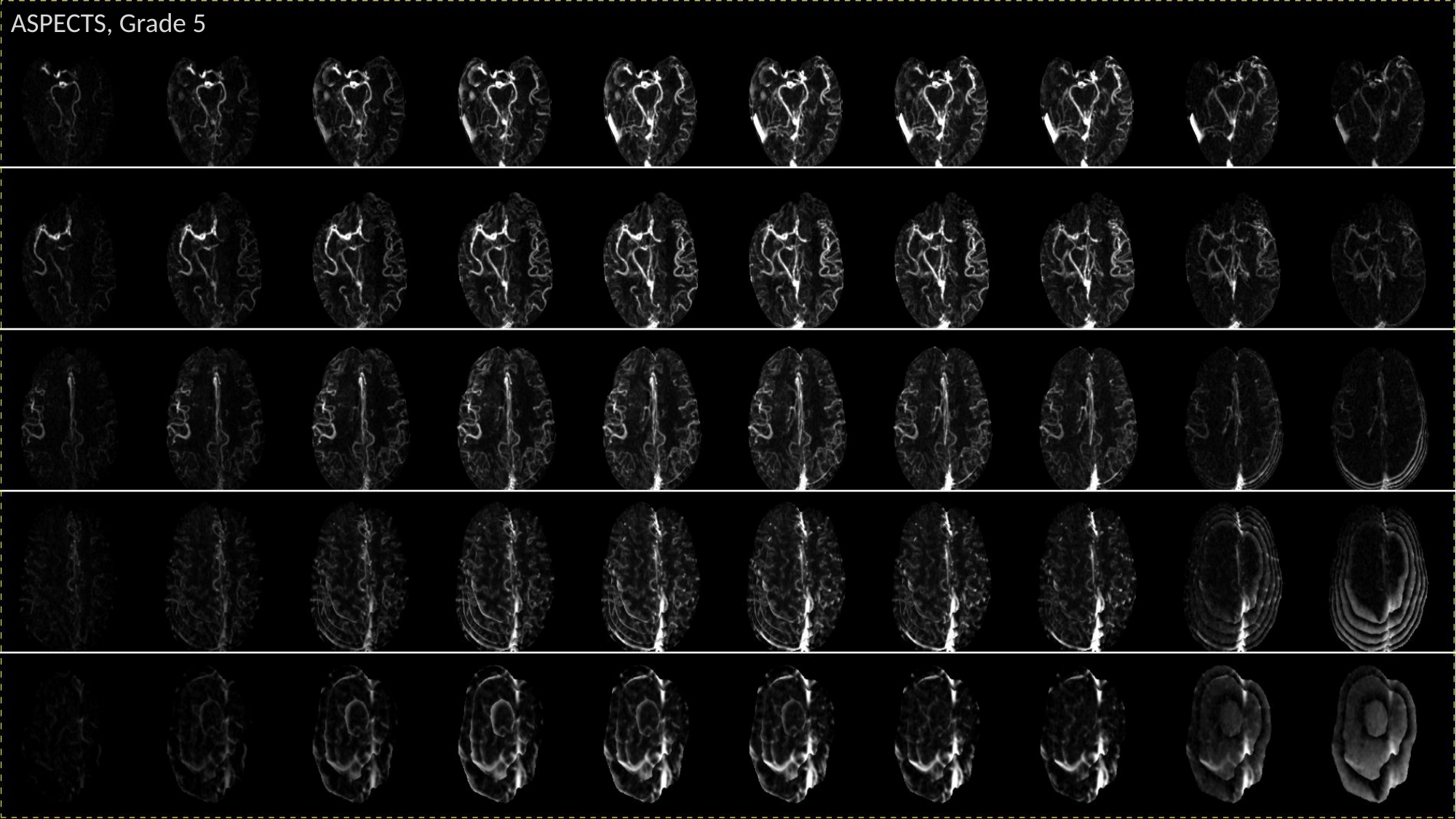

ASPECTS, Grade 5

## Slide 13
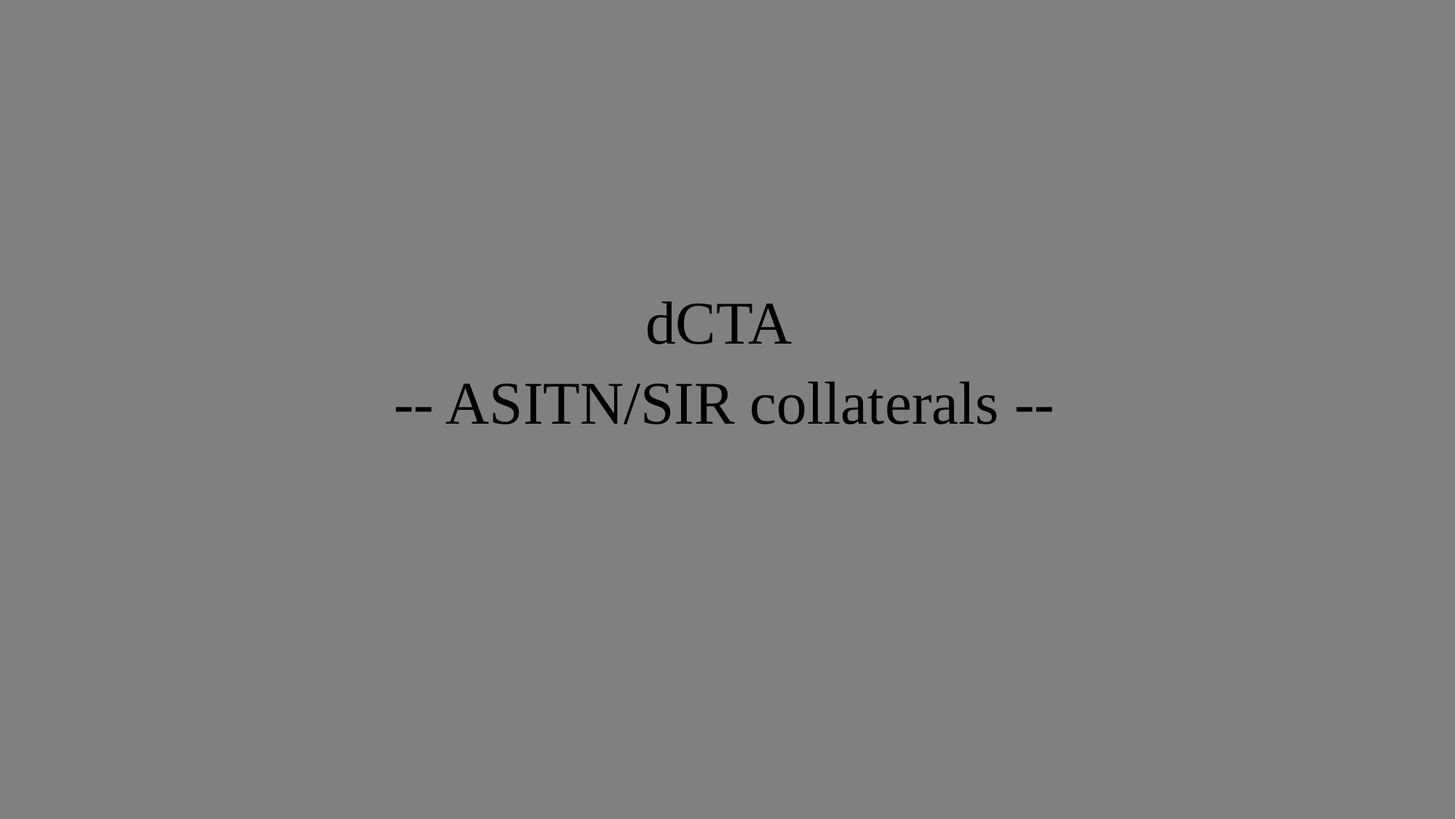

dCTA
-- ASITN/SIR collaterals --

## Slide 14
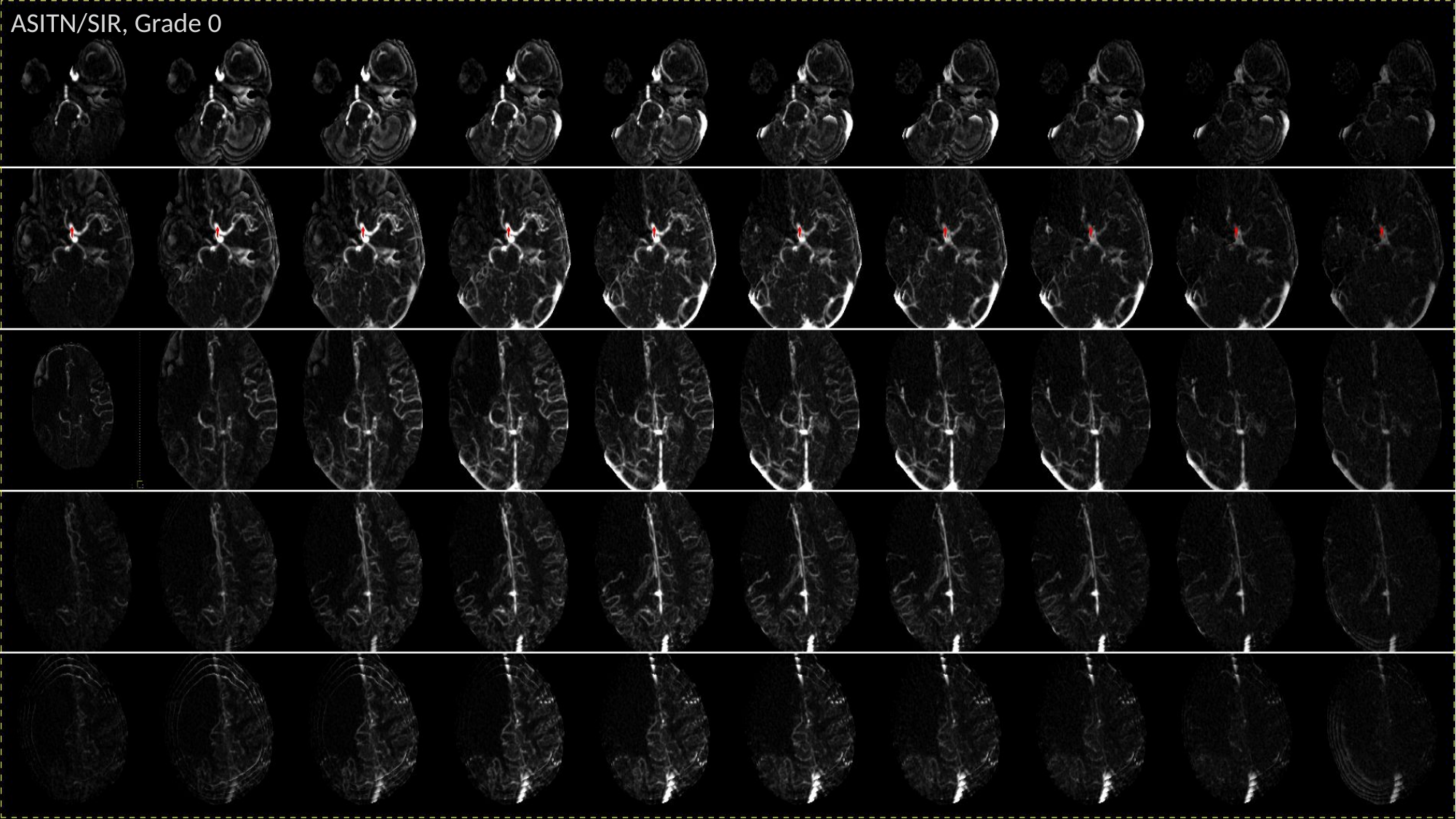

ASITN/SIR, Grade 0

## Slide 15
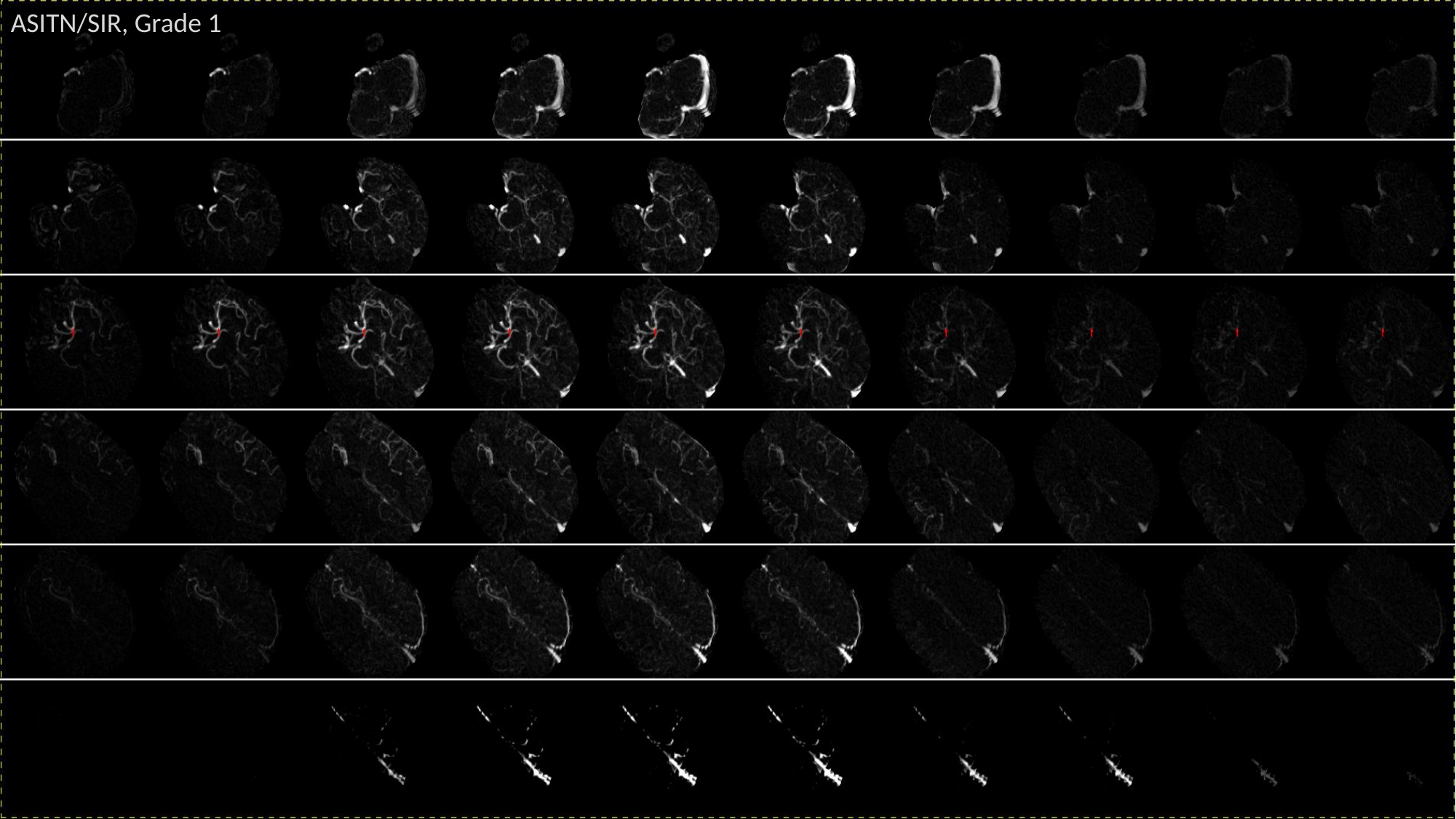

ASITN/SIR, Grade 1

## Slide 16
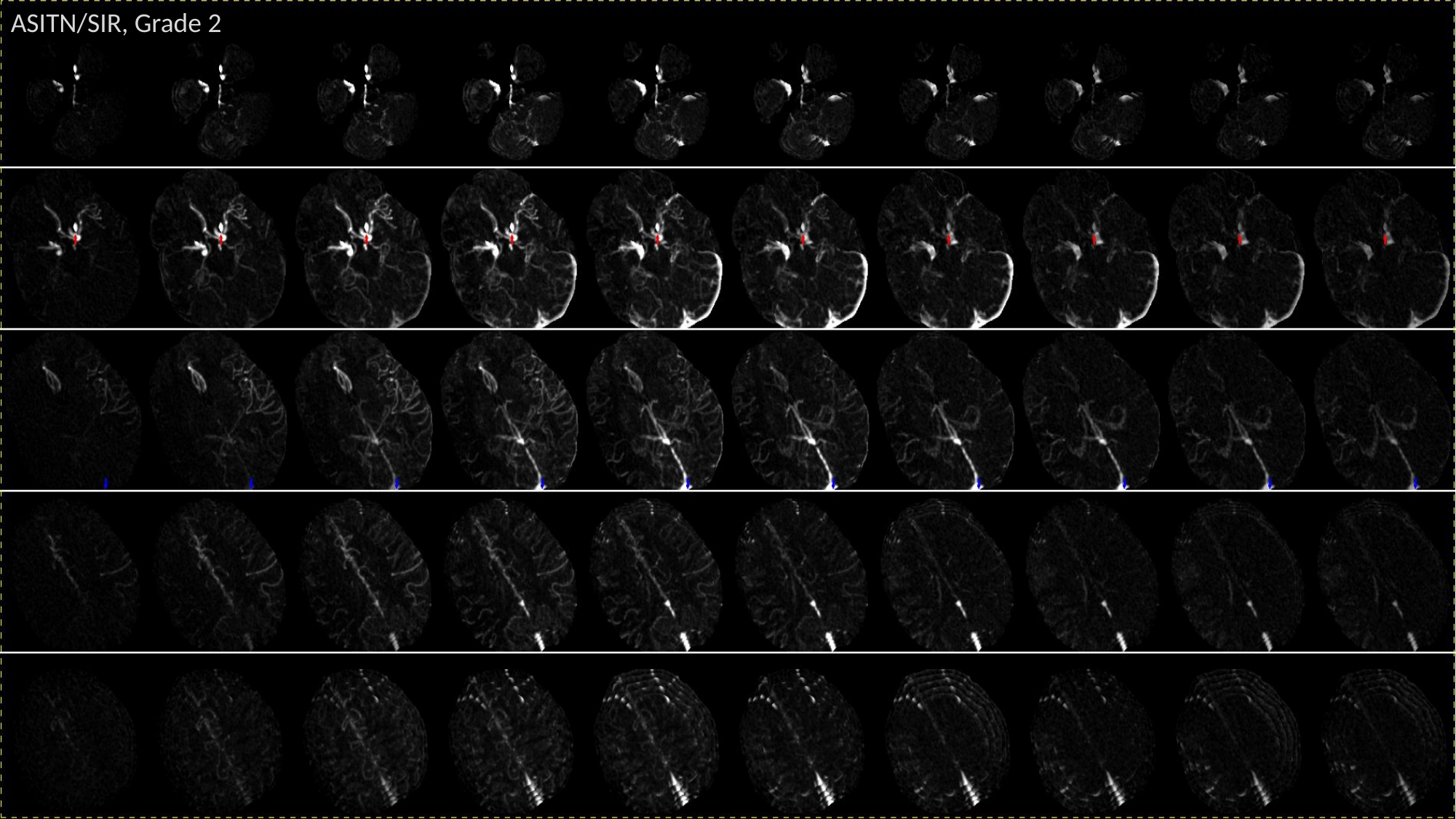

ASITN/SIR, Grade 2

## Slide 17
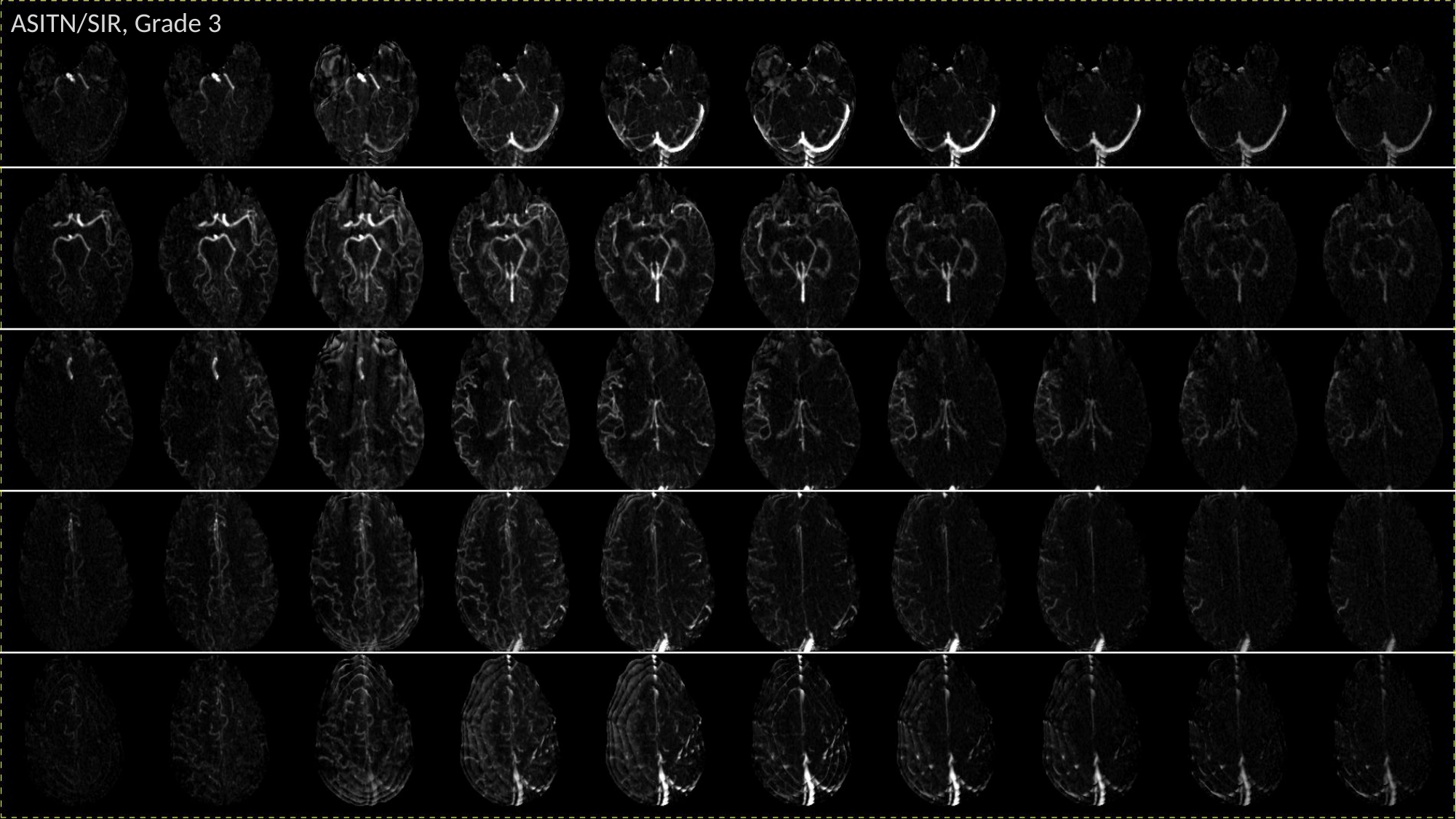

ASITN/SIR, Grade 3

## Slide 18
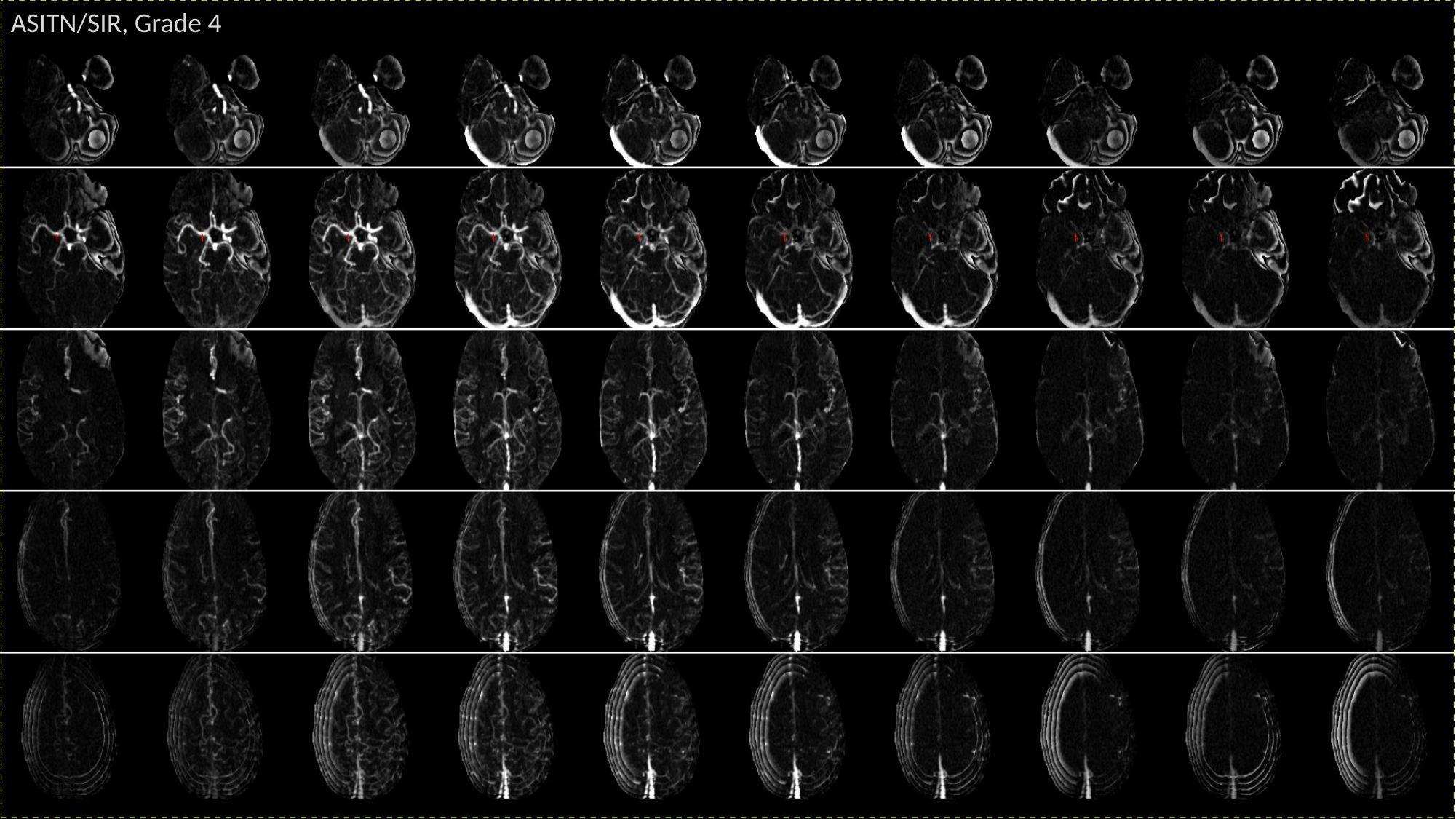

ASITN/SIR, Grade 4

## Slide 19
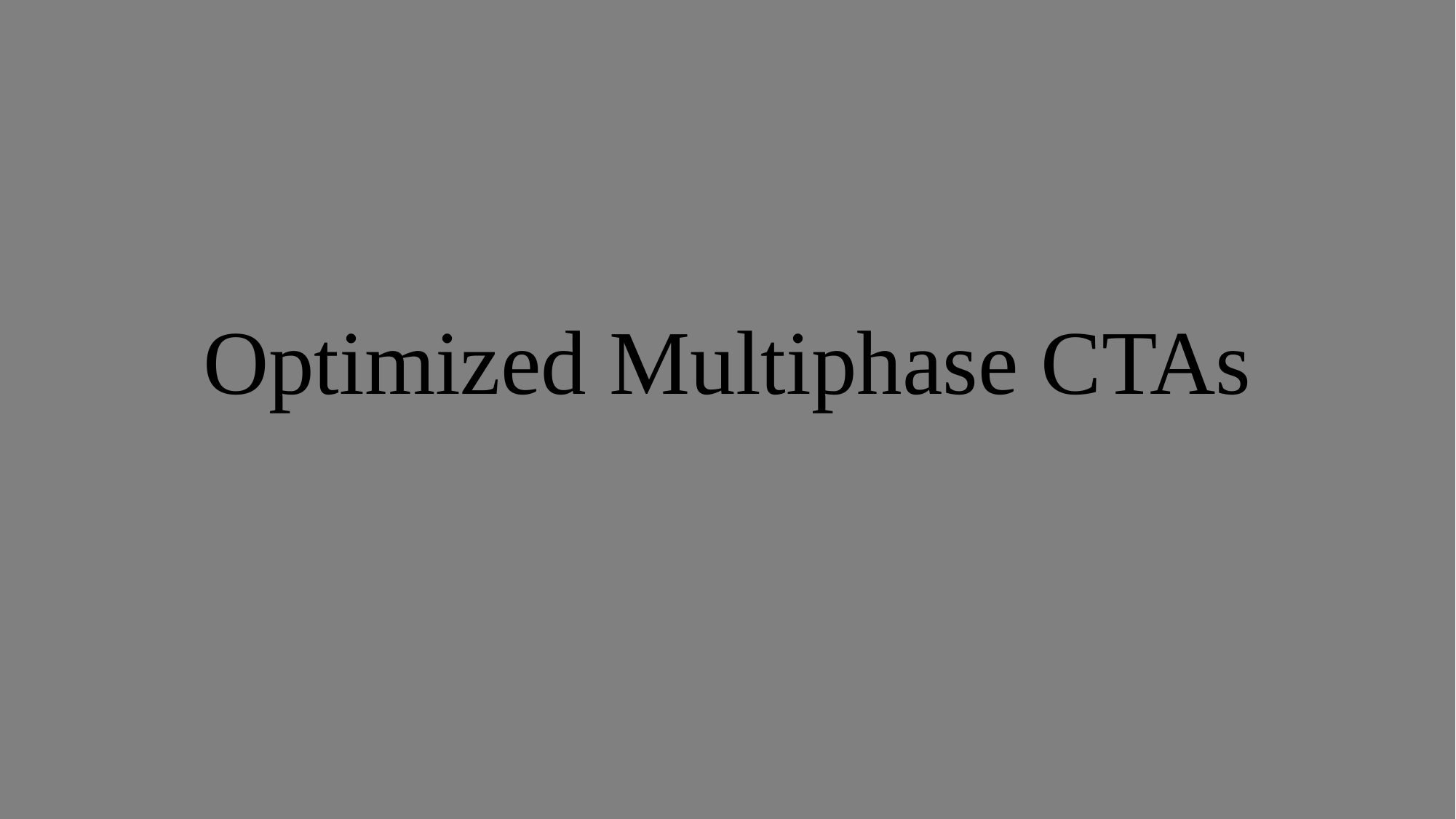

# Optimized Multiphase CTAs

## Slide 20
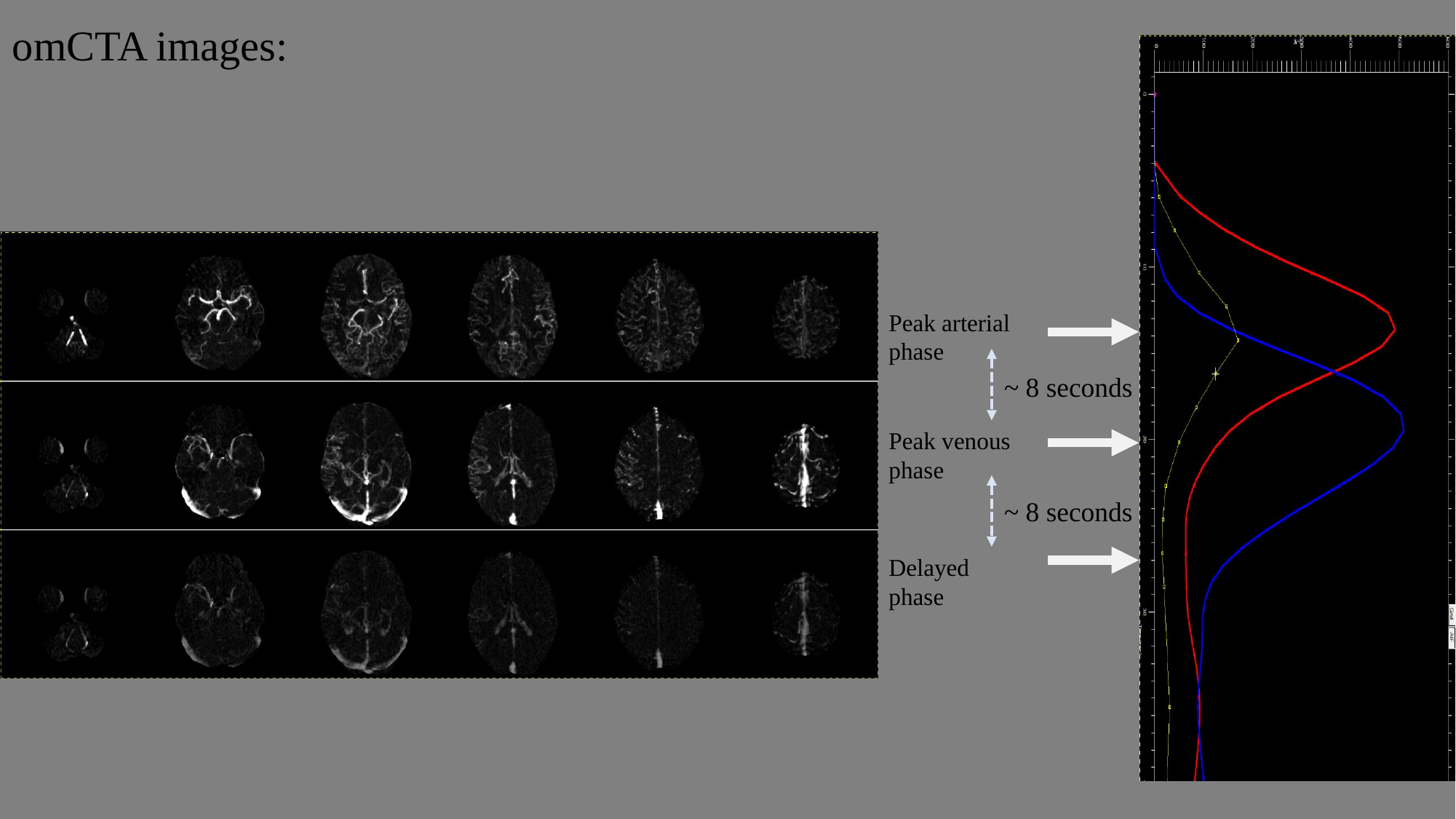

omCTA images:
Peak arterial phase
~ 8 seconds
Peak venous phase
~ 8 seconds
Delayed phase

## Slide 21
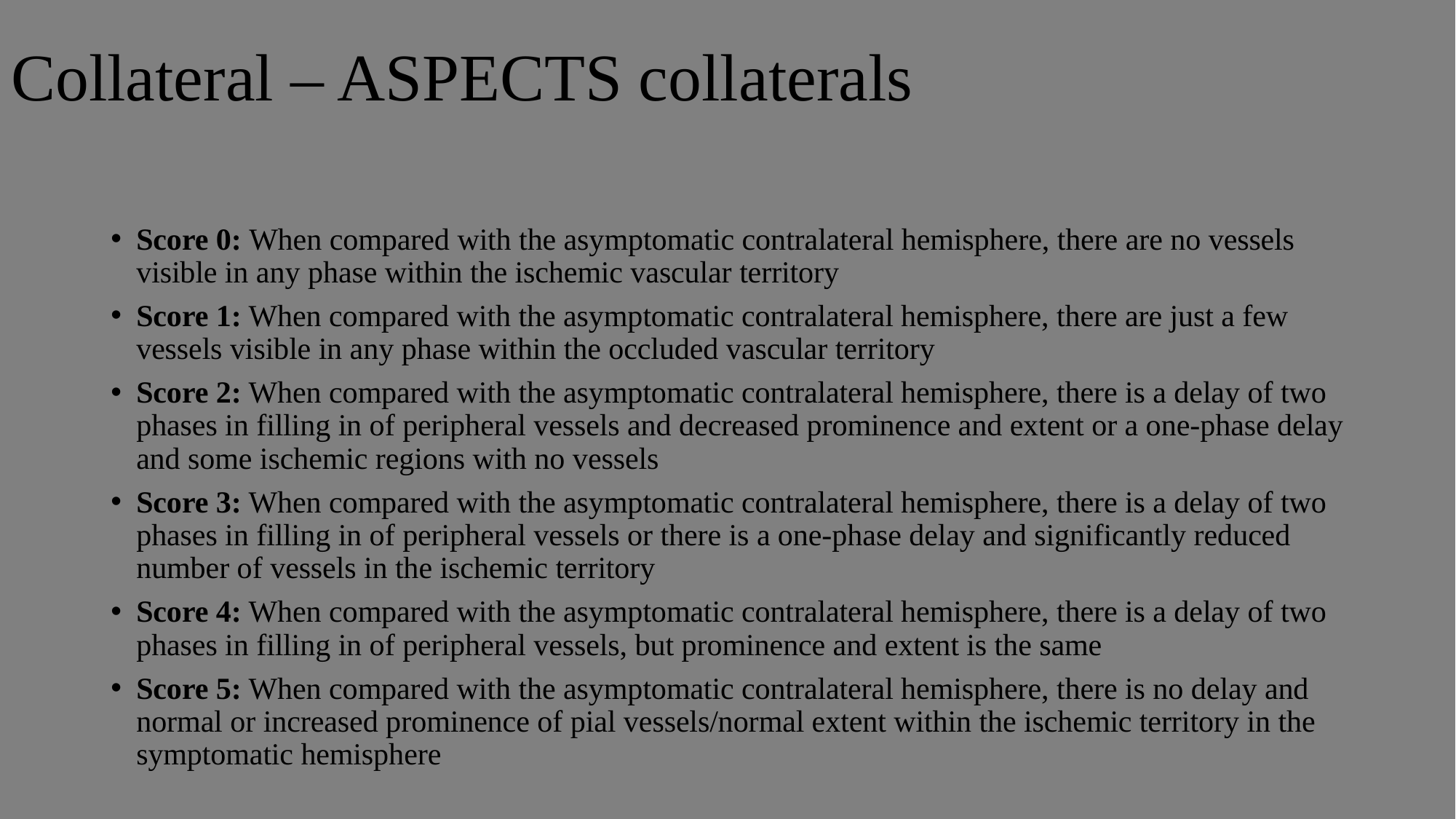

# Collateral – ASPECTS collaterals
Score 0: When compared with the asymptomatic contralateral hemisphere, there are no vessels visible in any phase within the ischemic vascular territory
Score 1: When compared with the asymptomatic contralateral hemisphere, there are just a few vessels visible in any phase within the occluded vascular territory
Score 2: When compared with the asymptomatic contralateral hemisphere, there is a delay of two phases in filling in of peripheral vessels and decreased prominence and extent or a one-phase delay and some ischemic regions with no vessels
Score 3: When compared with the asymptomatic contralateral hemisphere, there is a delay of two phases in filling in of peripheral vessels or there is a one-phase delay and significantly reduced number of vessels in the ischemic territory
Score 4: When compared with the asymptomatic contralateral hemisphere, there is a delay of two phases in filling in of peripheral vessels, but prominence and extent is the same
Score 5: When compared with the asymptomatic contralateral hemisphere, there is no delay and normal or increased prominence of pial vessels/normal extent within the ischemic territory in the symptomatic hemisphere

## Slide 22
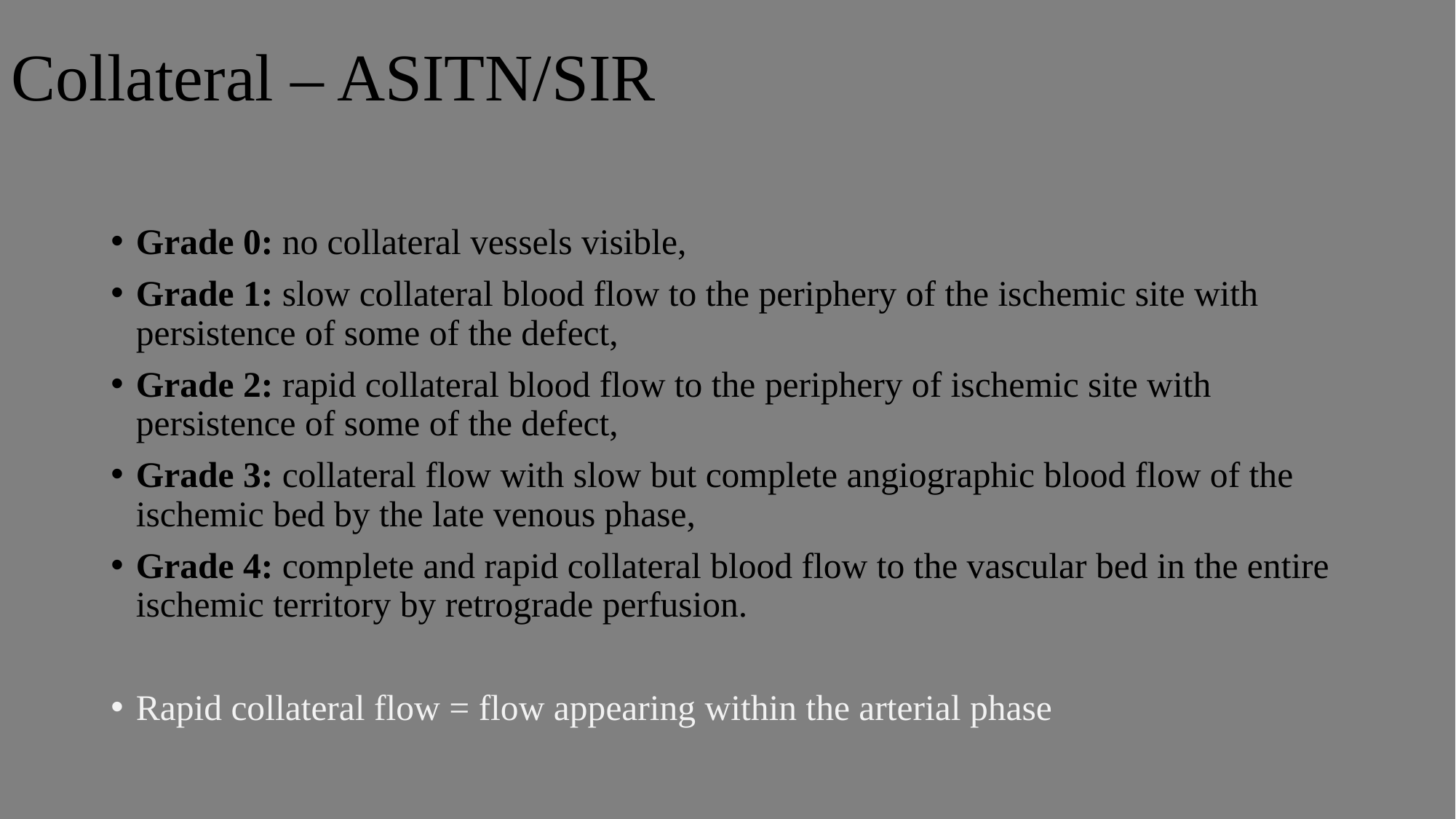

# Collateral – ASITN/SIR
Grade 0: no collateral vessels visible,
Grade 1: slow collateral blood flow to the periphery of the ischemic site with persistence of some of the defect,
Grade 2: rapid collateral blood flow to the periphery of ischemic site with persistence of some of the defect,
Grade 3: collateral flow with slow but complete angiographic blood flow of the ischemic bed by the late venous phase,
Grade 4: complete and rapid collateral blood flow to the vascular bed in the entire ischemic territory by retrograde perfusion.
Rapid collateral flow = flow appearing within the arterial phase

## Slide 23
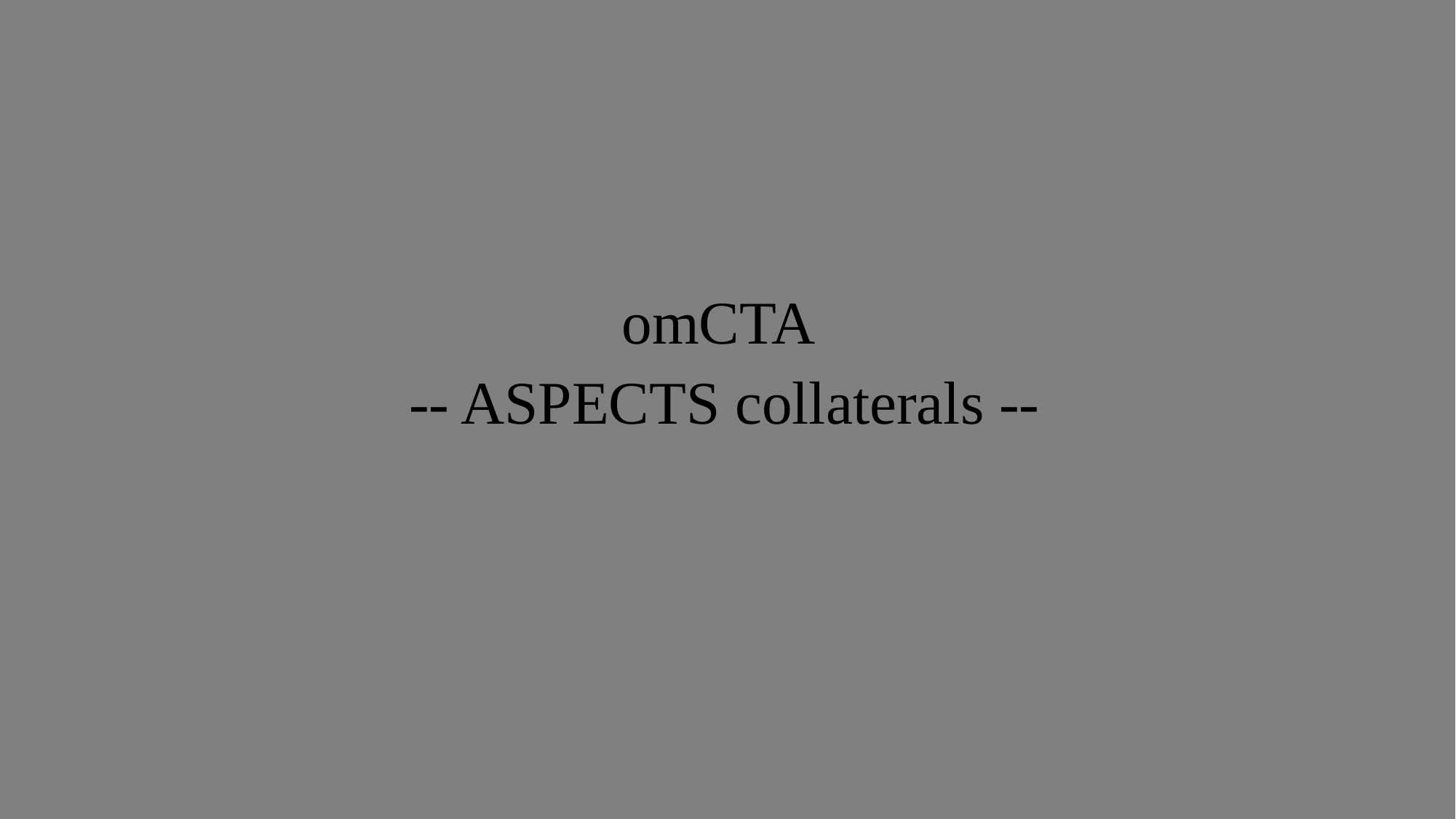

omCTA
-- ASPECTS collaterals --

## Slide 24
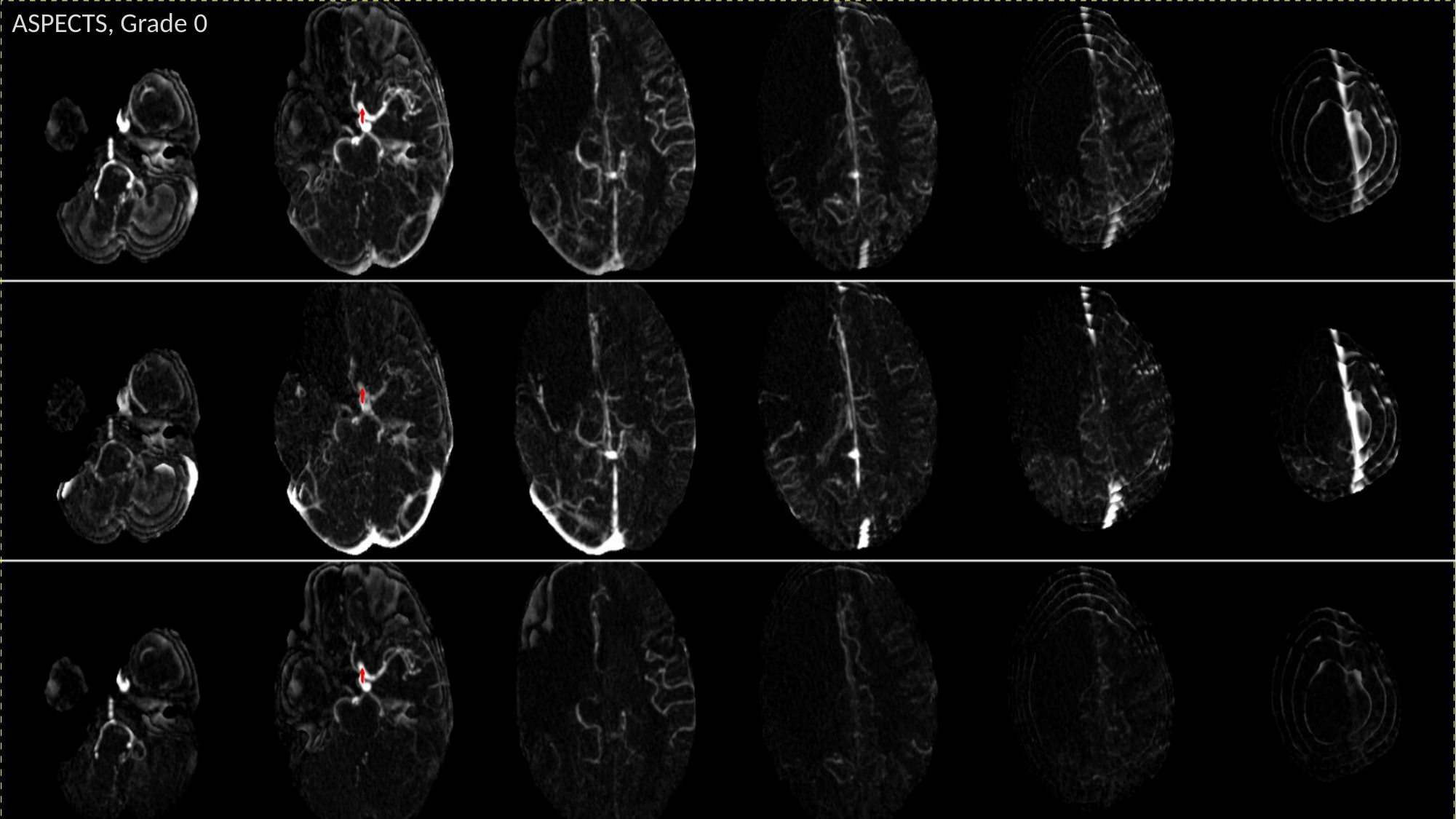

ASPECTS, Grade 0

## Slide 25
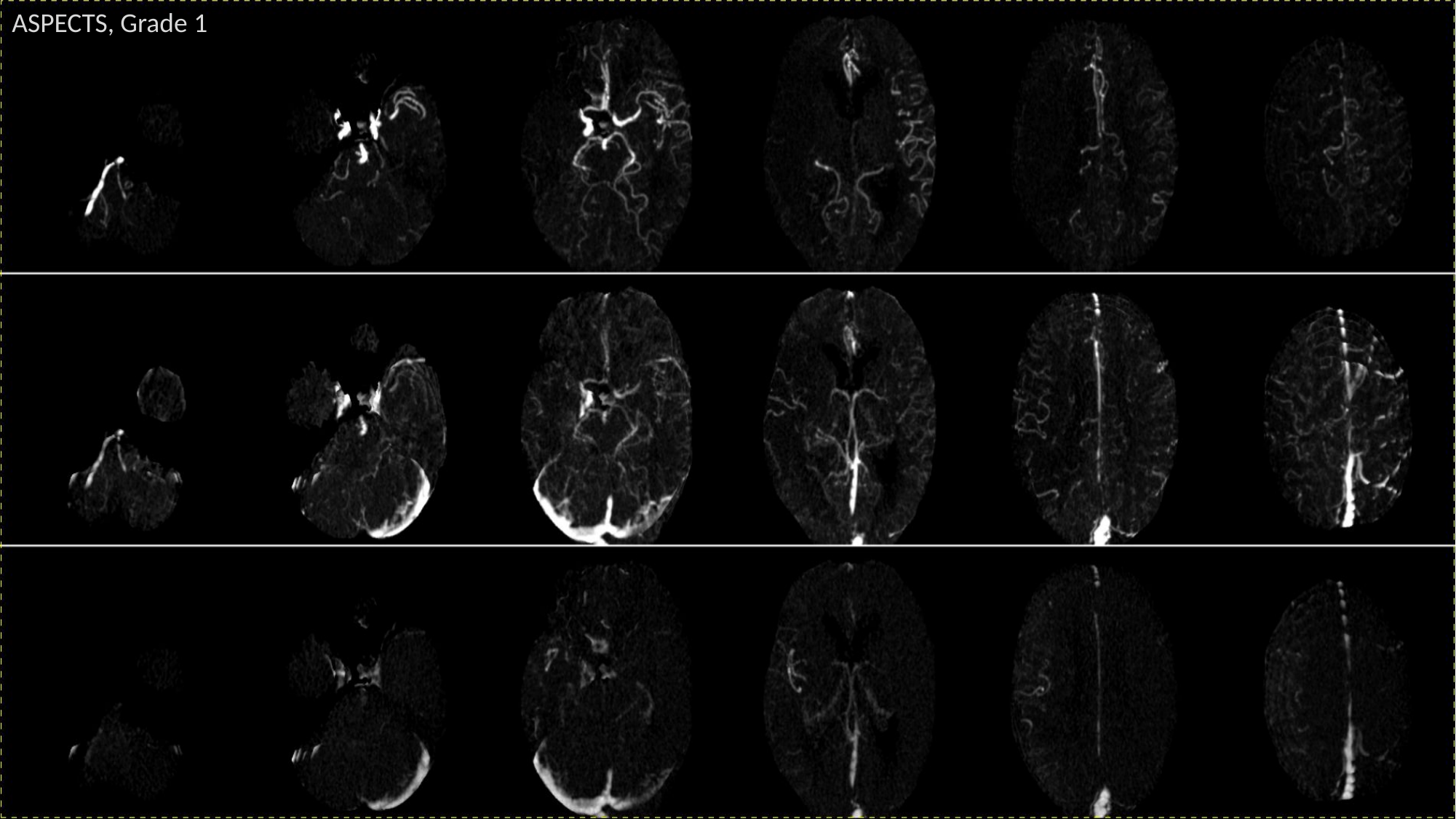

ASPECTS, Grade 1

## Slide 26
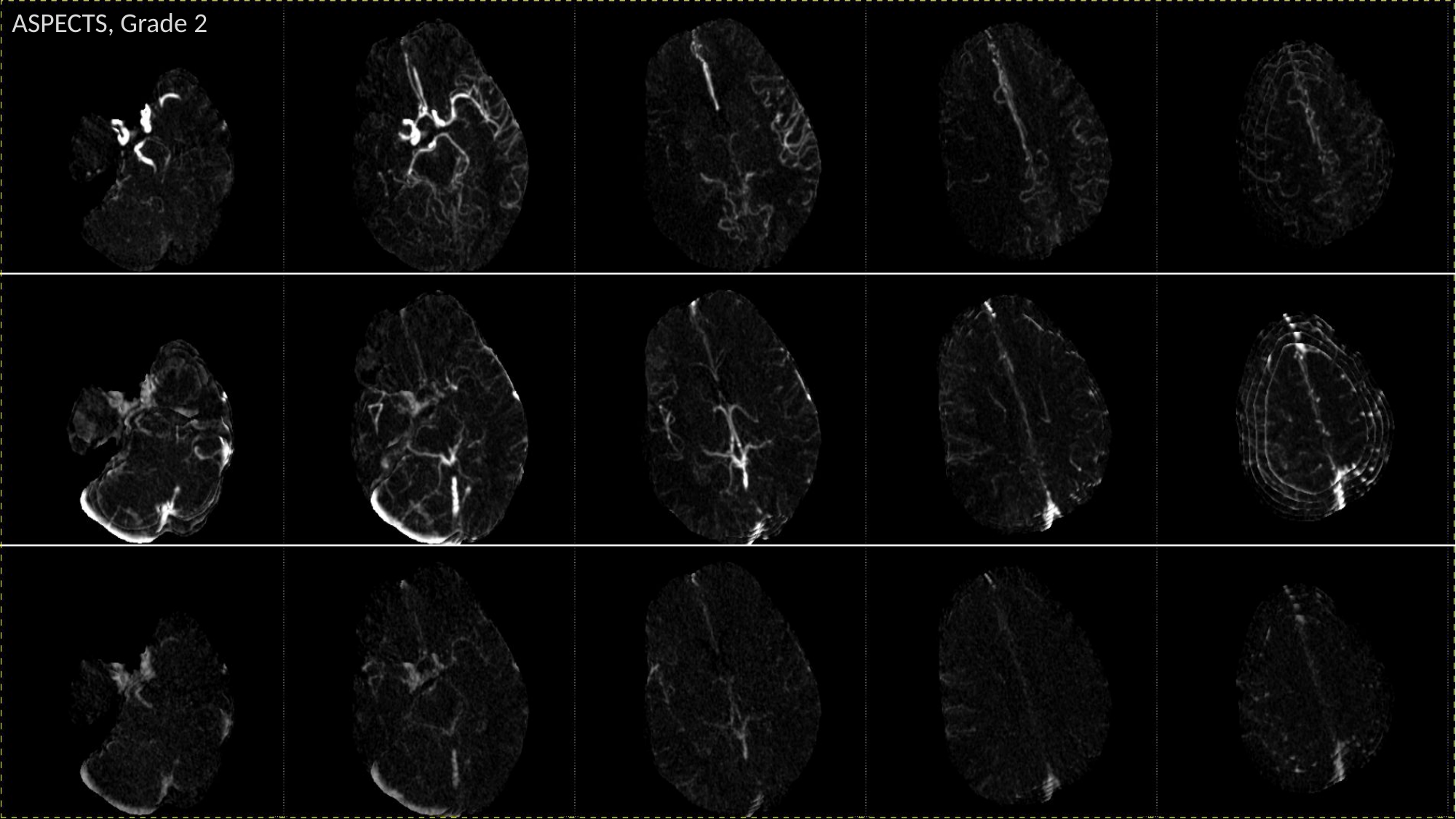

ASPECTS, Grade 2

## Slide 27
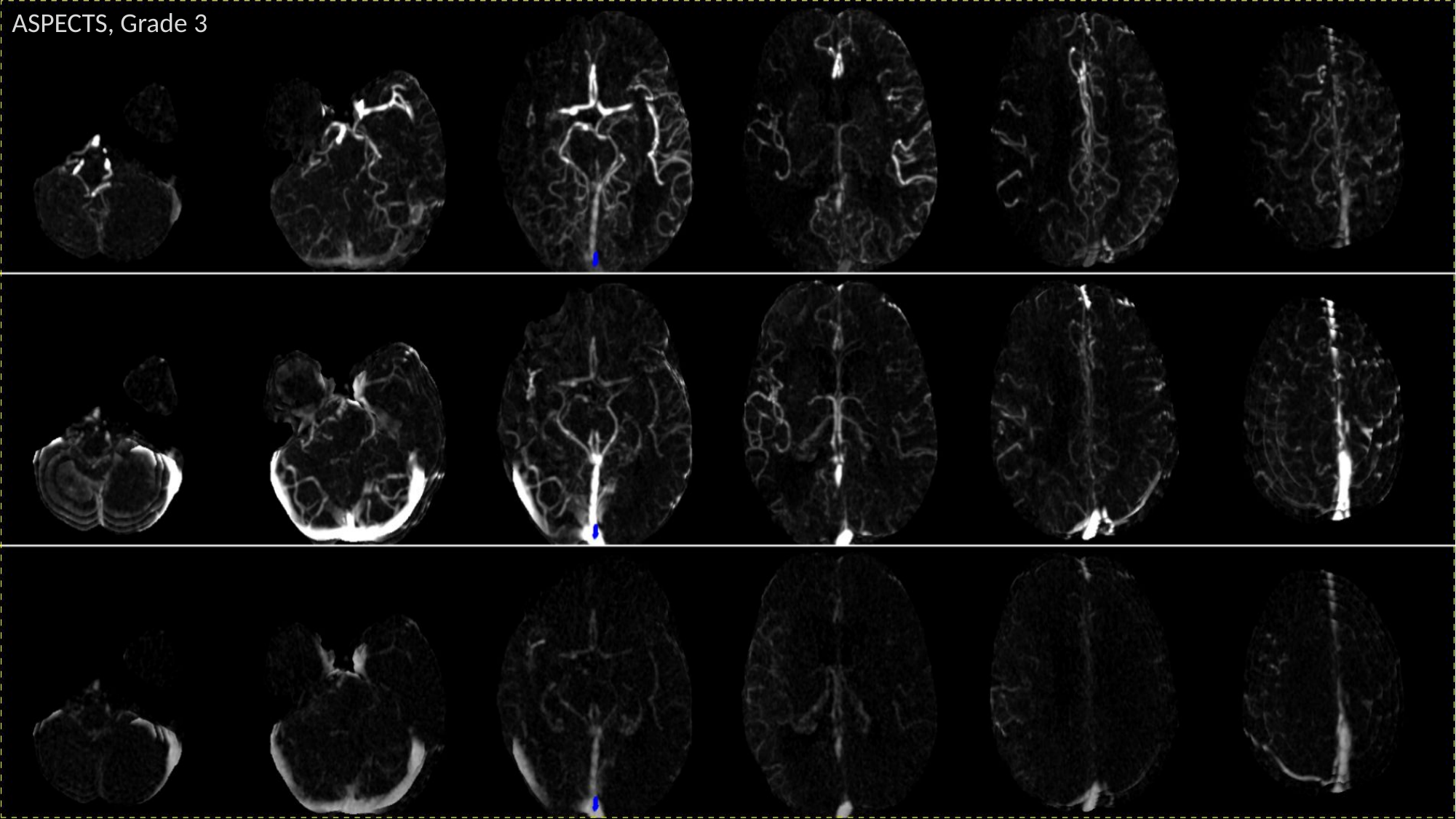

ASPECTS, Grade 3

## Slide 28
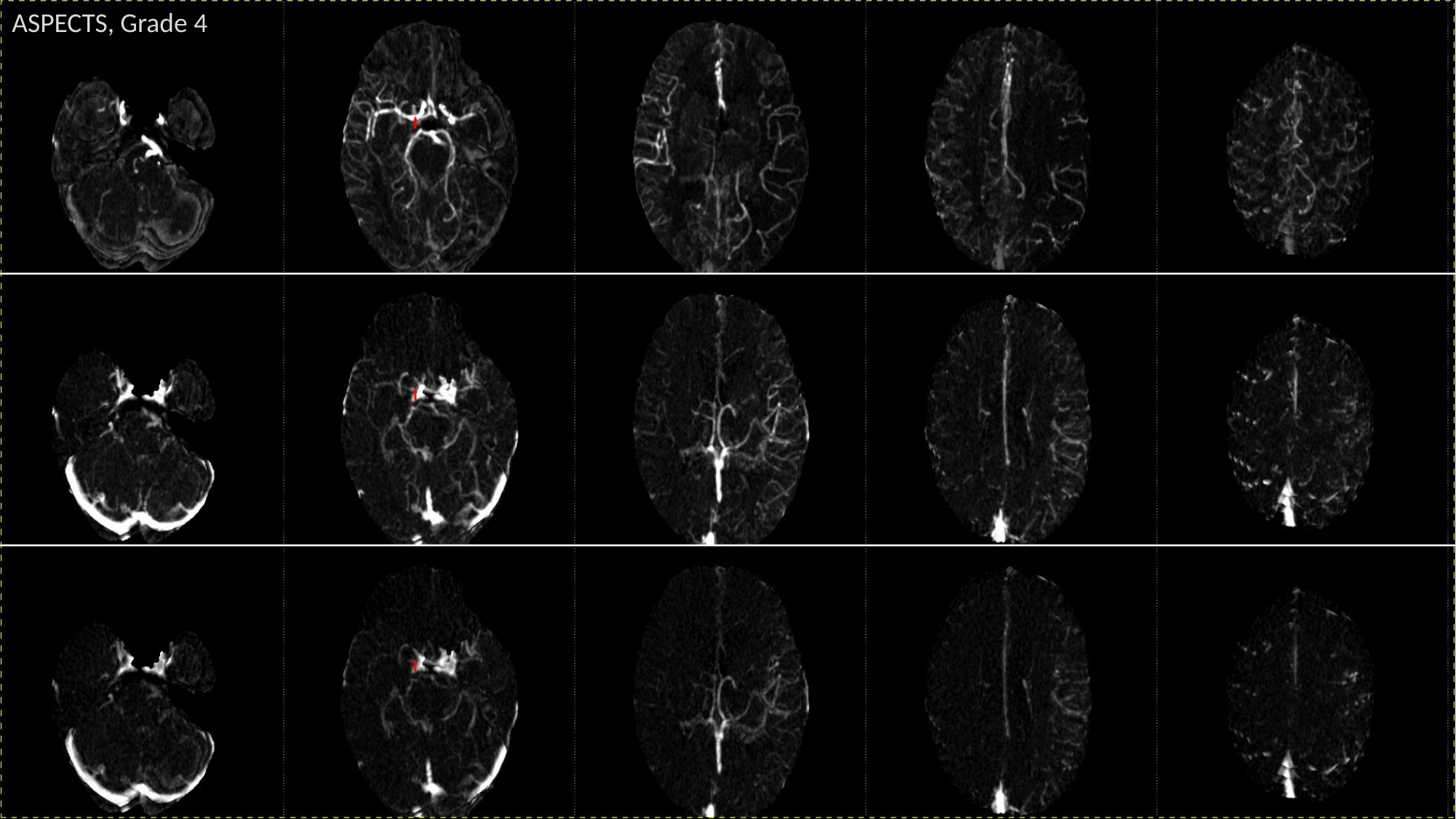

ASPECTS, Grade 4

## Slide 29
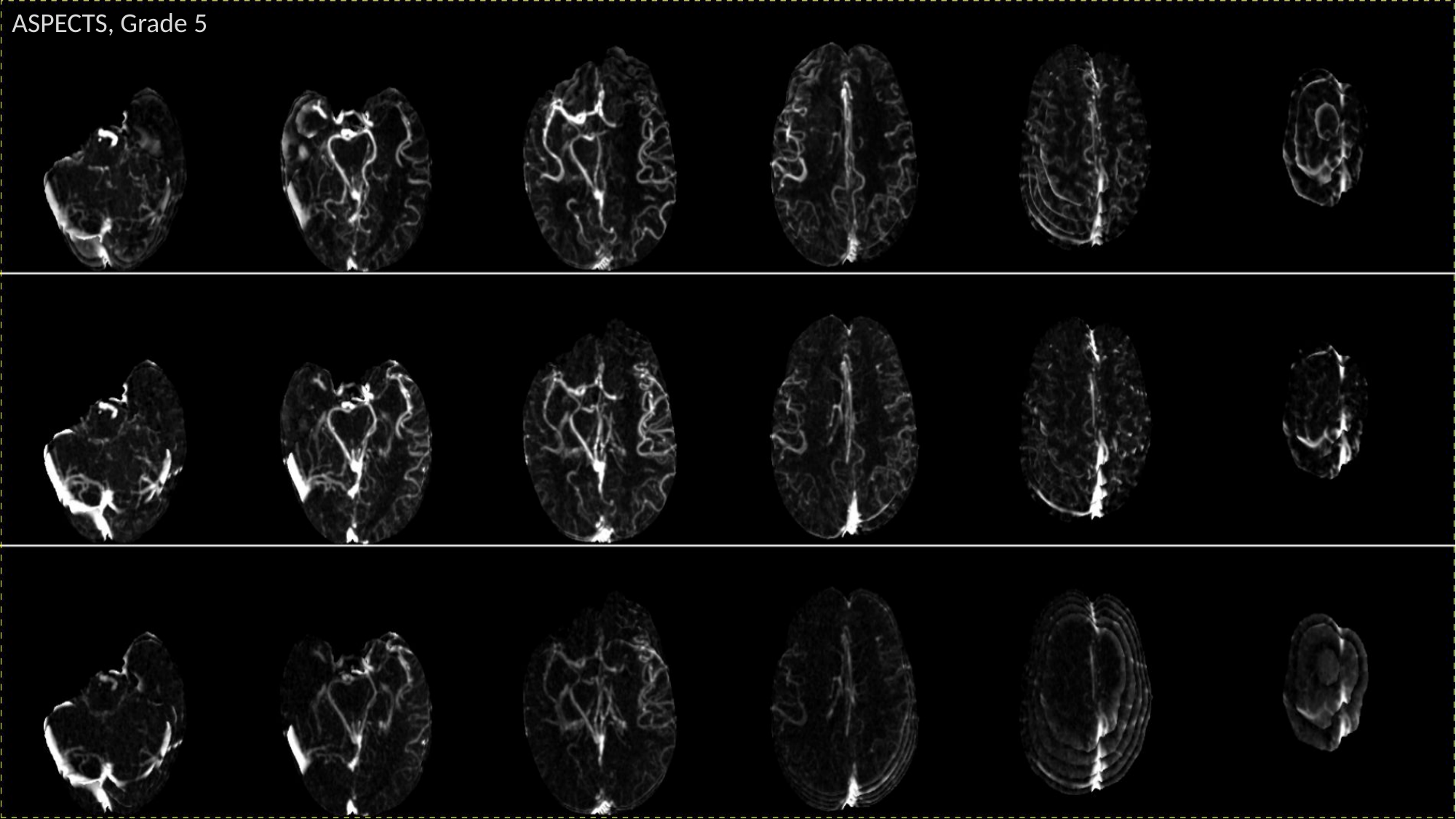

ASPECTS, Grade 5

## Slide 30
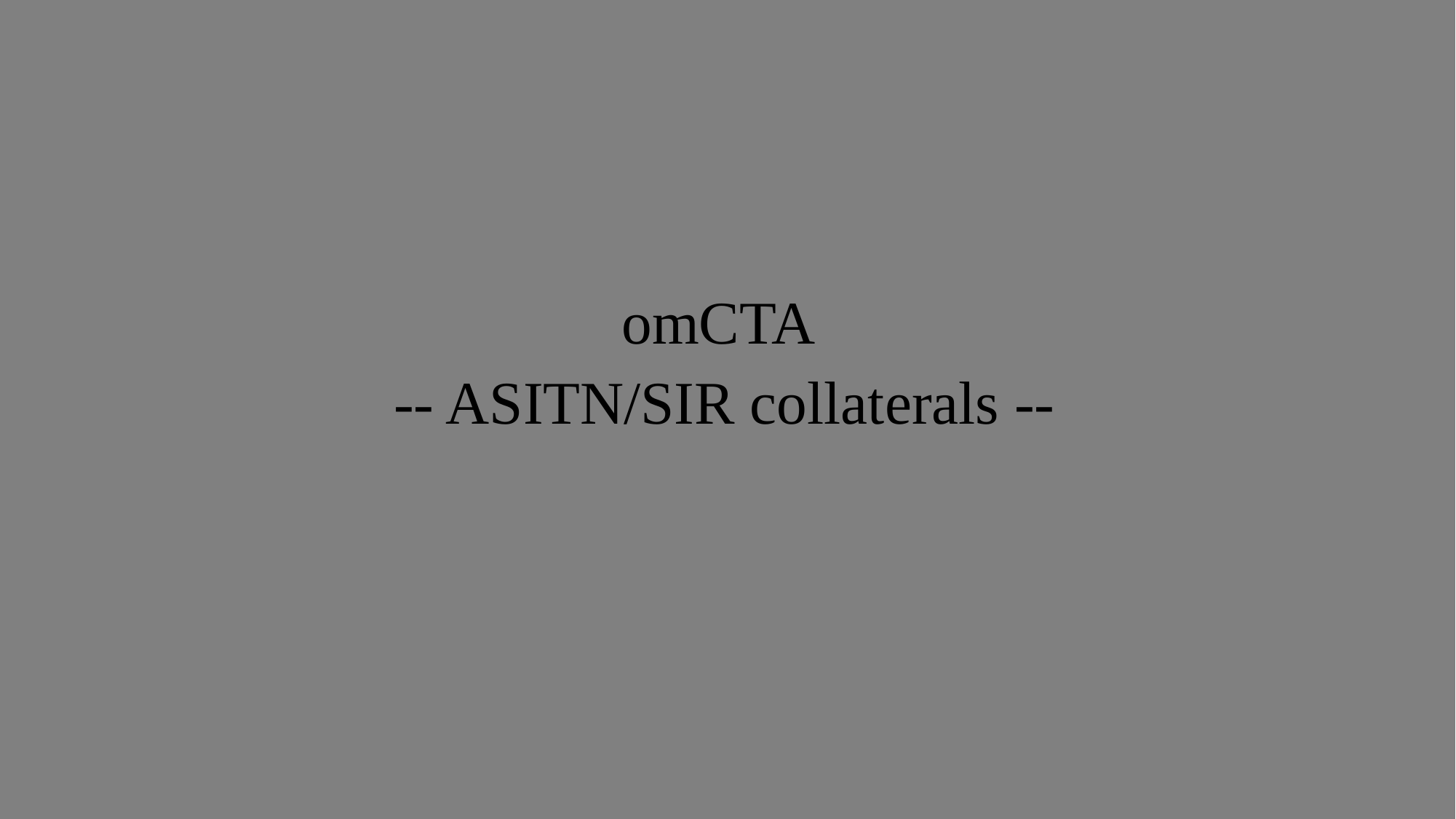

omCTA
-- ASITN/SIR collaterals --

## Slide 31
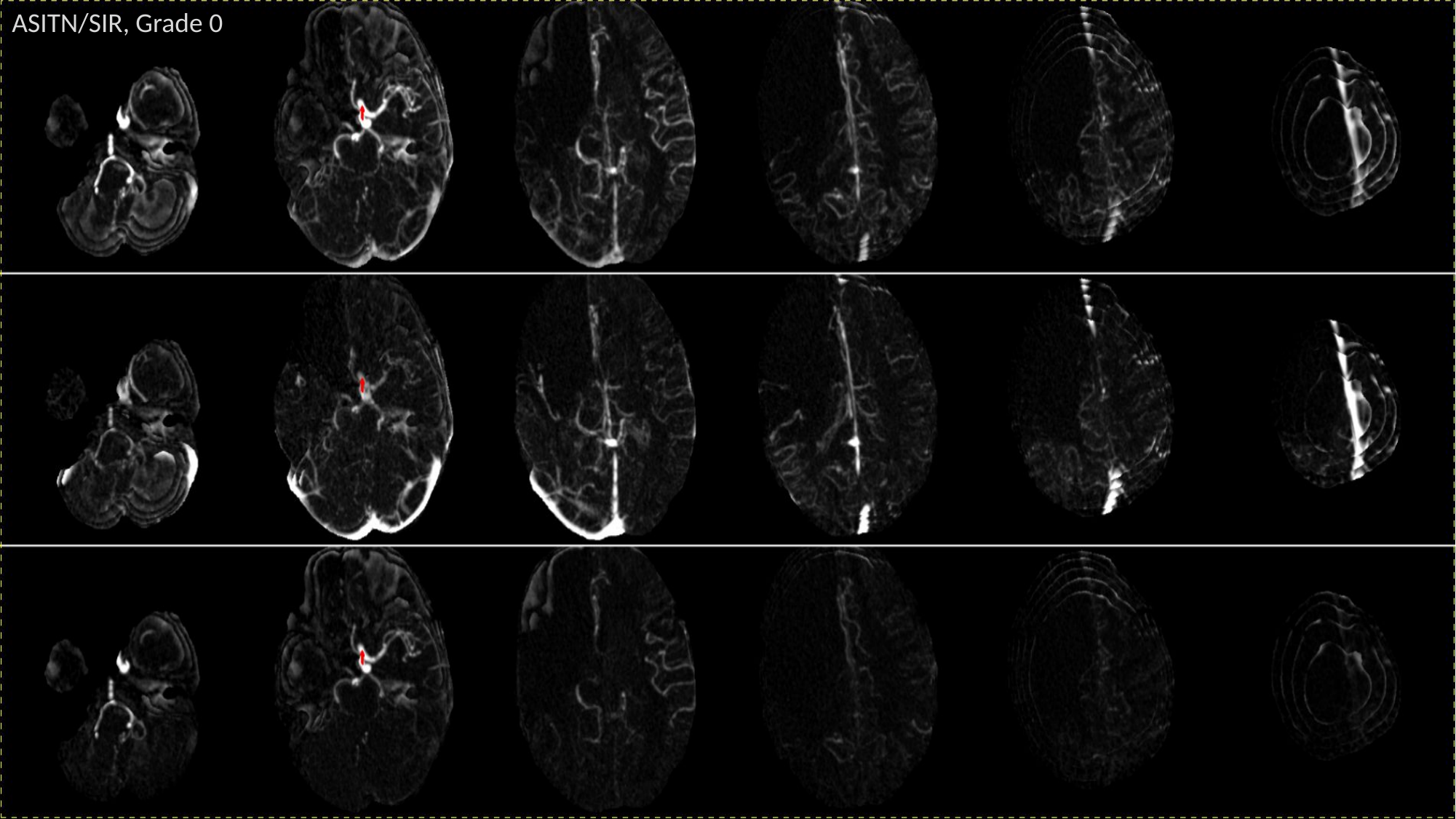

ASITN/SIR, Grade 0

## Slide 32
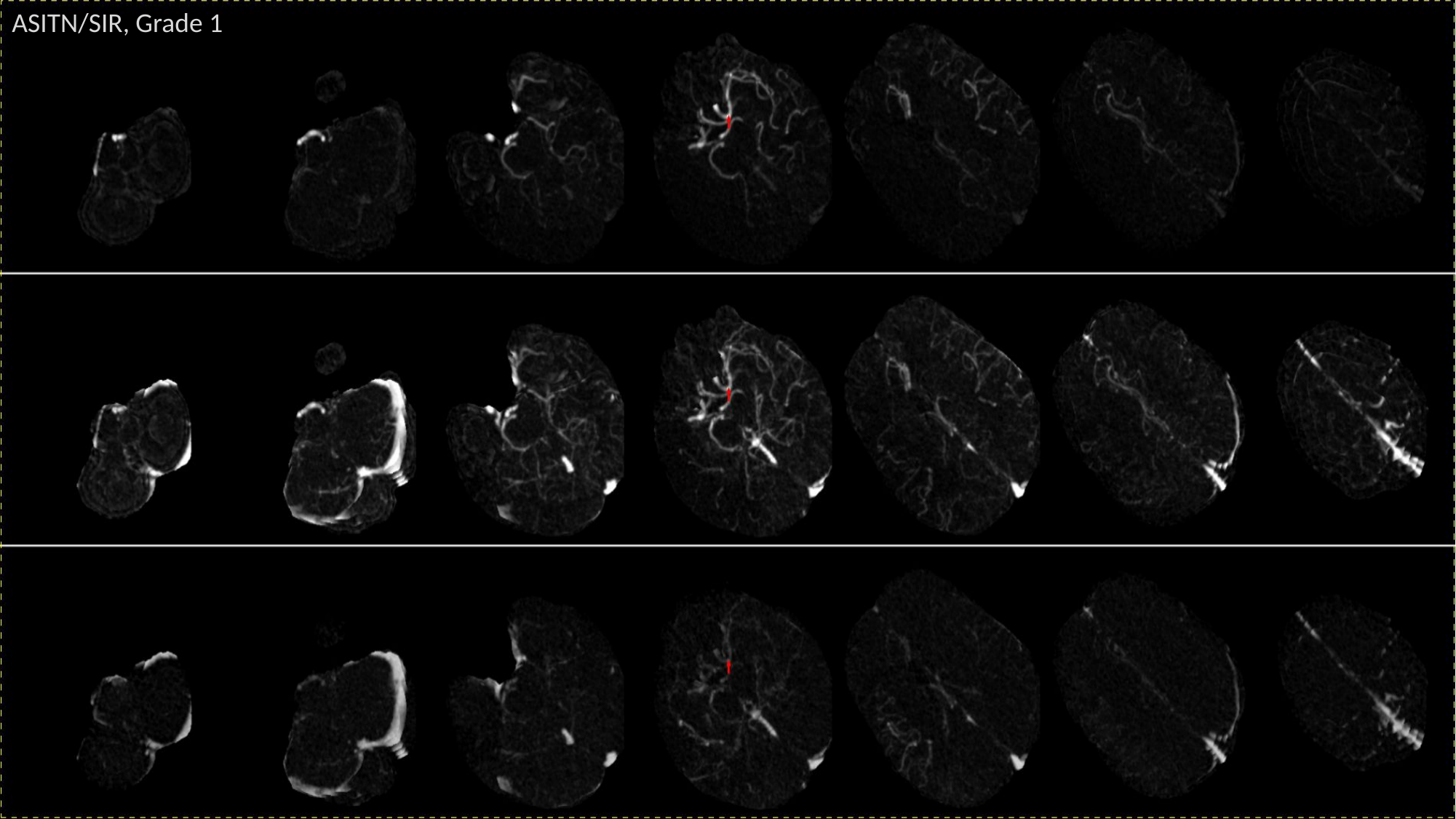

ASITN/SIR, Grade 1

## Slide 33
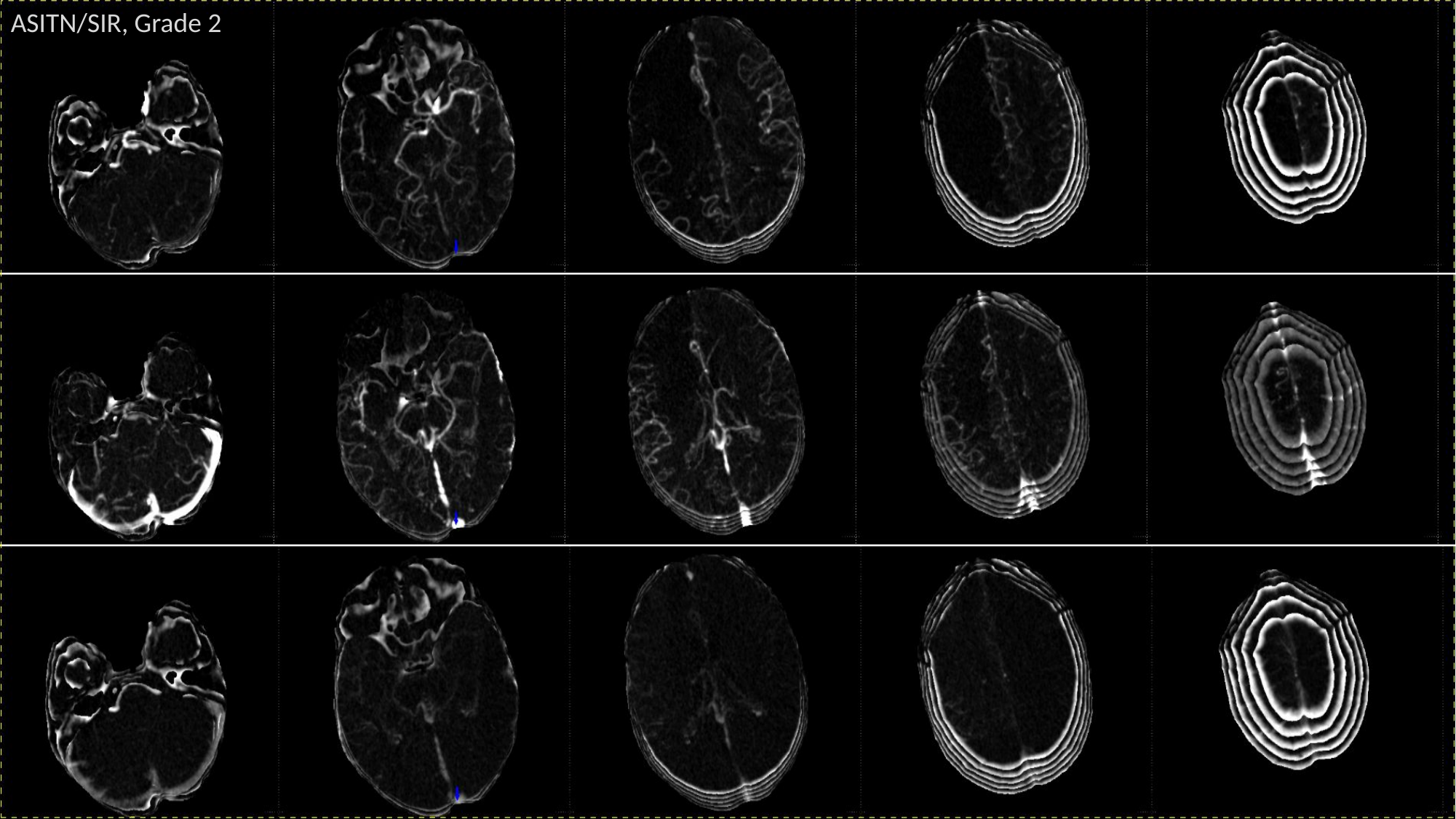

ASITN/SIR, Grade 2

## Slide 34
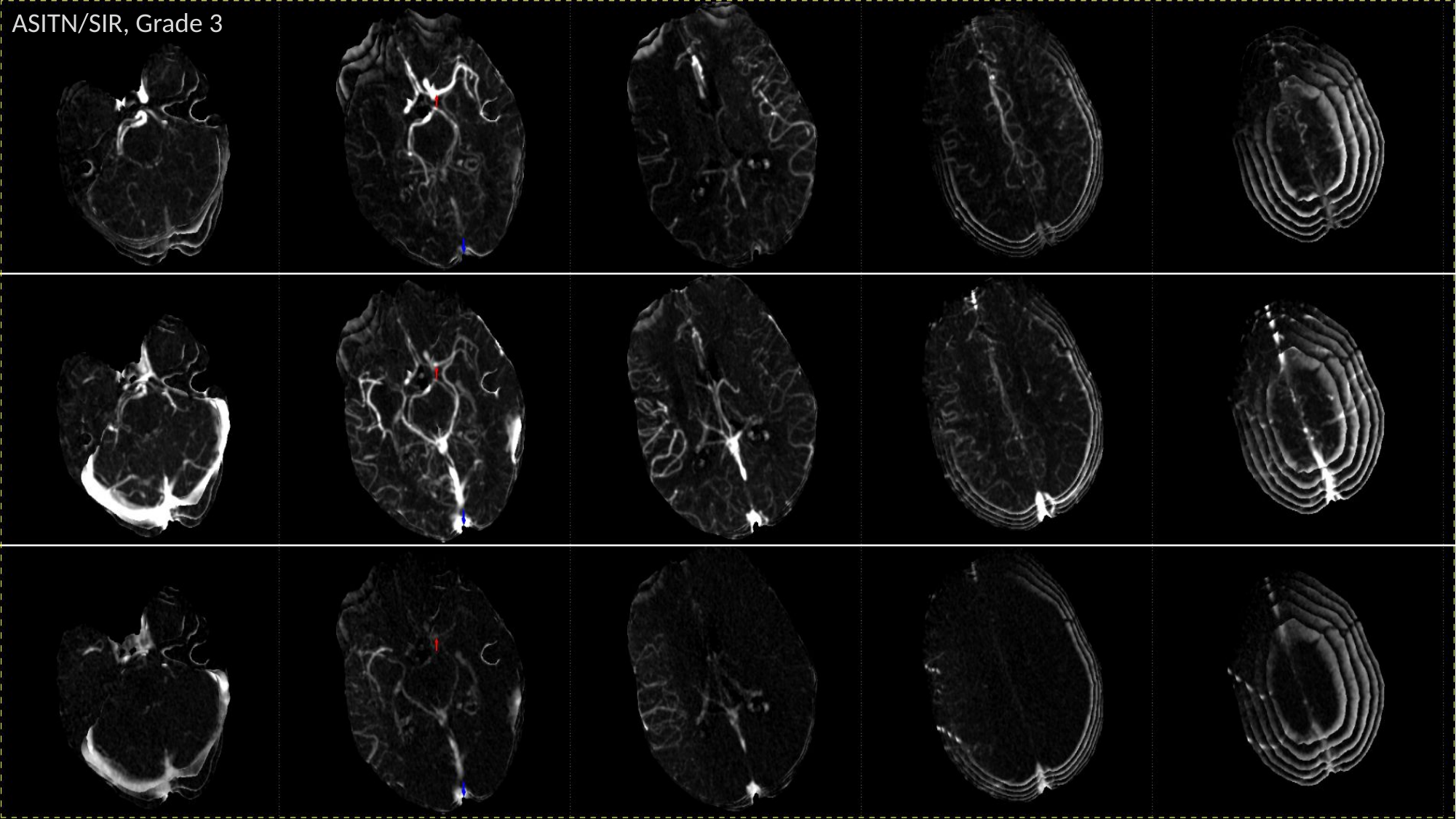

ASITN/SIR, Grade 3

## Slide 35
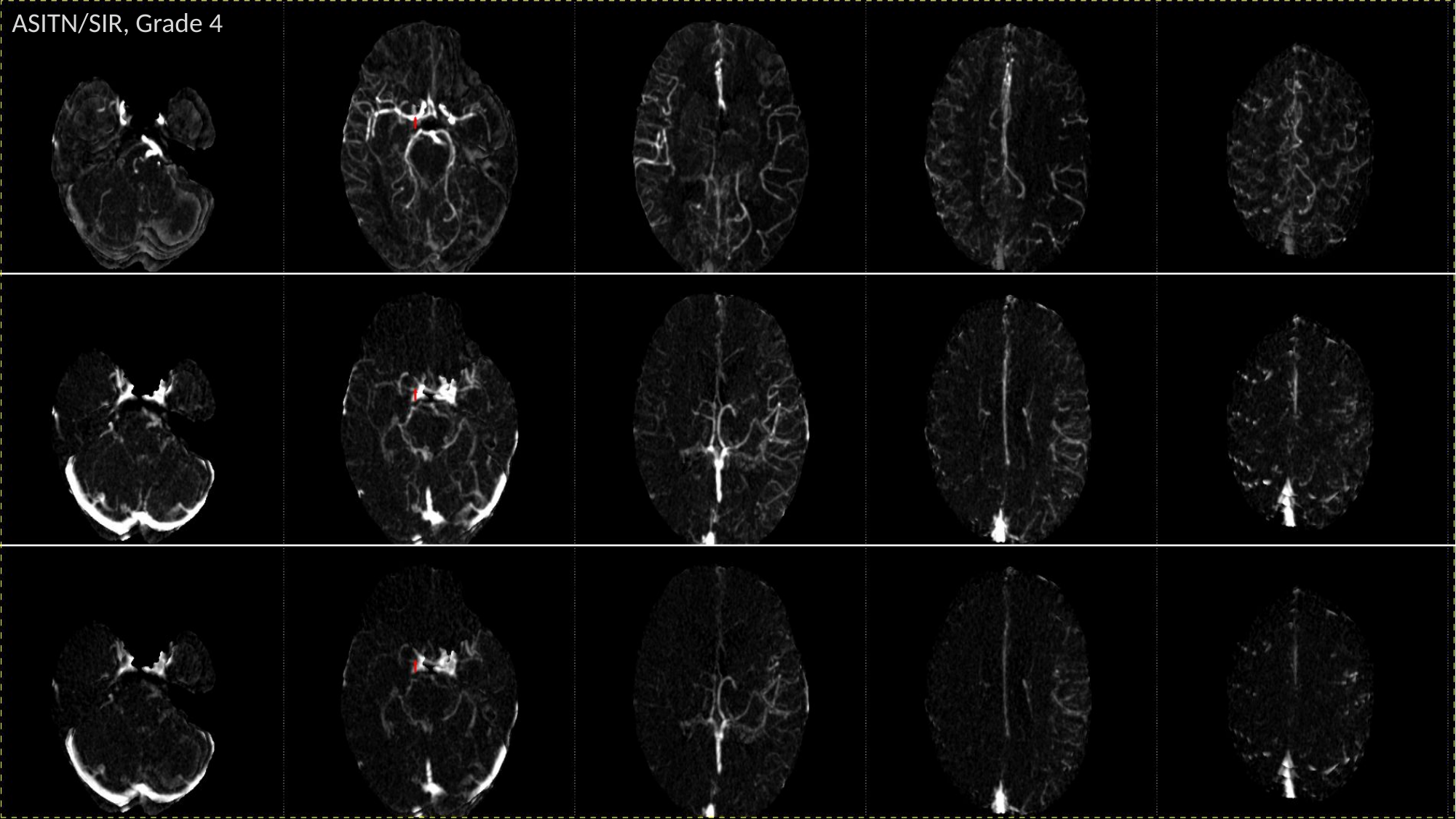

ASITN/SIR, Grade 4
